# Supplementary material for: Multivariate resilience indicators to anticipate vector-borne disease outbreaks: A West Nile virus case-study
Source: PLoS Comput Biol. 2025 Oct 13;21(10):e1012703. doi: 10.1371/journal.pcbi.1012703 (PMC12539738; doi:10.1371/journal.pcbi.1012703)
Supplement: S1 Text — (PDF) [file pcbi.1012703.s001.pdf]

Supporting Information

Model details

Equations

Model equations are based on a simplified version of the model provided in (Laperriere et al., 2011).

$$\frac{\mathrm{dM_s}}{\mathrm{dt}} = b_M N_M - \mathrm{p}_M k \frac{p_{BV} B_{VI} + p_{BH} B_{HI}}{p_{BV} N_{BV} + p_{BH} N_{BH} + p_H N_H + p_E N_E} M_S - b_M M_S$$

$$\frac{dM_E}{dt} = p_M k \frac{p_{BV} B_{VI} + p_{BH} B_{HI}}{p_{BV} N_{BV} + p_{BH} N_{BH} + p_H N_H + p_E N_E} M_S - \gamma_E M_E - b_M M_E$$

$$\frac{dM_I}{dt} = \gamma_E M_E - b_M M_I$$

$$\frac{dB_{VS}}{dt} = b_B N_{BV} - p_M k \frac{p_{BV} M_I}{p_{BV} N_{BV} + p_{BH} N_{BH} + p_H N_H + p_E N_E} B_{VS} - b_B B_{VS}$$

$$\frac{dB_{VE}}{dt} = p_M k \frac{p_{BV} M_I}{p_{BV} N_{BV} + p_{BH} N_{BH} + p_H N_H + p_E N_E} B_{VS} - \gamma_B B_{VE} - b_B B_{VE}$$

$$\frac{dB_{VI}}{dt} = \gamma_B B_{VE} - \alpha_B B_{VI} - b_B B_{VI}$$

$$\frac{dB_{VR}}{dt} = (1 - \nu_{BV}) \alpha_B B_{VI} - b_B B_{VR}$$

$$\frac{dB_{HS}}{dt} = b_B N_{BH} - p_M k \frac{p_{BH} M_I}{p_{BV} N_{BV} + p_{BH} N_{BH} + p_H N_H + p_E N_E} B_{HS} - b_B B_{HS}$$

$$\frac{dB_{HE}}{dt} = p_M k \frac{p_{BH} M_I}{p_{BV} N_{BV} + p_{BH} N_{BH} + p_H N_H + p_E N_E} B_{HS} - \gamma_B B_{HE} - b_B B_{HE}$$

$$\frac{dB_{HI}}{dt} = \gamma_B B_{HE} - \alpha_B B_{HI} - b_B B_{HI}$$

$$\frac{dB_{HR}}{dt} = (1 - \nu_{BH}) \alpha_B B_{HI} - b_B B_{HR}$$

$$\frac{dH_S}{dt} = - p_M k \frac{p_H M_I}{p_{BV} N_{BV} + p_{BH} N_{BH} + p_H N_H + p_E N_E} H_S$$

$$\frac{dH_E}{dt} = p_M k \frac{p_H M_I}{p_{BV} N_{BV} + p_{BH} N_{BH} + p_H N_H + p_E N_E} H_S - \gamma_H H_E$$

$$\frac{dH_I}{dt} = \gamma_H H_E - \alpha_H H_I$$

$$\frac{H_R}{dt} = \alpha_H H_I$$

$$\frac{dE_S}{dt} = b_E N_E - \ p_M k \frac{p_E M_I}{p_{BV} N_{BV} + p_{BH} N_{BH} + p_H N_H + p_E N_E} E_S - b_E E_S$$

$$\frac{dE_E}{dt} = \ p_M k \frac{p_E M_I}{p_{BV} N_{BV} + p_{BH} N_{BH} + p_H N_H + p_E N_E} E_S - \gamma_E E_E - b_E E_E$$

$$\frac{dE_I}{dt} = \gamma_E E_E - \alpha_E E_I - b_E E_I$$

$$\frac{dE_R}{dt} = (1 - \nu_E) \alpha_E E_I - b_E E_R$$

Parameter values

Table A: Parameter values for the model. Parameters are taken from (de Wit et al., 2024; Laperriere et al., 2011).

Parameter	Notation	Value	Unit
N_m	Mosquito abundance	300 000	-
N_{BV}	Bird V (visible) abundance	200 000	-
N_{BH}	Bird H (hidden) abundance	200 000	-
N_H	Human abundance	2 000 000	-
N_E	Horse abundance	40 000	-
b_M	Birth/death rate mosquitoes	0.22	$.day^{-1}$
γ_B	Incubation rate birds	0.55	$.day^{-1}$
γ_H	Incubation rate humans	0.25	$.day^{-1}$
γ_E	Incubation rate horses	0.05	$.day^{-1}$
γ_M	Incubation rate mosquitoes	0.11	$.day^{-1}$
ν_{BV}	Disease induced death-rate bird V	0.3	$.day^{-1}$
ν_{BH}	Disease induced death-rate bird H	0.05	$.day^{-1}$
ν_E	Disease induced death-rate horses	0.04	$.day^{-1}$
α_B	Recovery rate birds	0.31	$.day^{-1}$
α_H	Recovery rate humans	0.0714	$.day^{-1}$
α_E	Recovery rate horses	0.2	$.day^{-1}$
b_B	Birth/death rate birds	0.002	$.day^{-1}$
b_E	Birth/death rate horses	0.00016	$.day^{-1}$
Rate_import	Importation rate of infected birds	0.15	$.day^{-1}$
p_{BV}	Mosquito feeding preference towards bird V	5	-
p_{BH}	Mosquito feeding preference towards bird H	10 (initially)	-
p_H	Mosquito feeding preference towards humans	1	-
p_E	Mosquito feeding preference towards horses	1	-
p_M	Transmission probability	0.9	-
k	Biting rate	Varying	$.day^{-1}$

Next Generation matrix

The Next Generation matrix was used to estimate R_0 as well as the typical infection coefficient calculated in the eigenvector associated with the dominant eigenvalue of the NGM. The NGM with large domain is calculated numerically using the formula provided in (Diekmann et al., 2010): $K_L = -T\ \Sigma^{-1}$, with Σ being the transition matrix and T the transmission matrix. T and Σ are defined as follows for our system:

$$T = \begin{pmatrix}
0 & 0 & 0 & p_M k \frac{p_{Bv}}{p_{Bv}N_{Bv} + p_{BH}N_{BH} + p_H N_H + p_E N_E} N_M & 0 & p_M k \frac{p_{BH}}{p_{Bv}N_{Bv} + p_{BH}N_{BH} + p_H N_H + p_E N_E} N_M & 0 & 0 & 0 & 0 \\
0 & 0 & 0 & 0 & 0 & 0 & 0 & 0 & 0 & 0 \\
0 & p_M k \frac{p_{Bv}}{p_{Bv}N_{Bv} + p_{BH}N_{BH} + p_H N_H + p_E N_E} N_{BV} & 0 & 0 & 0 & 0 & 0 & 0 & 0 & 0 \\
0 & 0 & 0 & 0 & 0 & 0 & 0 & 0 & 0 & 0 \\
0 & p_M k \frac{p_{BH}}{p_{Bv}N_{Bv} + p_{BH}N_{BH} + p_H N_H + p_E N_E} N_{BH} & 0 & 0 & 0 & 0 & 0 & 0 & 0 & 0 \\
0 & 0 & 0 & 0 & 0 & 0 & 0 & 0 & 0 & 0 \\
0 & p_M k \frac{p_H}{p_{Bv}N_{Bv} + p_{BH}N_{BH} + p_H N_H + p_E N_E} N_H & 0 & 0 & 0 & 0 & 0 & 0 & 0 & 0 \\
0 & 0 & 0 & 0 & 0 & 0 & 0 & 0 & 0 & 0 \\
0 & p_M k \frac{p_E}{p_{Bv}N_{Bv} + p_{BH}N_{BH} + p_H N_H + p_E N_E} N_E & 0 & 0 & 0 & 0 & 0 & 0 & 0 & 0 \\
0 & 0 & 0 & 0 & 0 & 0 & 0 & 0 & 0 & 0
\end{pmatrix}$$

$$\Sigma = \begin{pmatrix}
-(\gamma_M + b_M) & 0 & 0 & 0 & 0 & 0 & 0 & 0 & 0 & 0 \\
\gamma_M & -b_M & 0 & 0 & 0 & 0 & 0 & 0 & 0 & 0 \\
0 & 0 & -(\gamma_B + b_B) & 0 & 0 & 0 & 0 & 0 & 0 & 0 \\
0 & 0 & \gamma_B & -(b_B + \alpha_B) & 0 & 0 & 0 & 0 & 0 & 0 \\
0 & 0 & 0 & 0 & -(\gamma_B + b_B) & 0 & 0 & 0 & 0 & 0 \\
0 & 0 & 0 & 0 & \gamma_B & -(b_B + \alpha_B) & 0 & 0 & 0 & 0 \\
0 & 0 & 0 & 0 & 0 & 0 & -\gamma_H & 0 & 0 & 0 \\
0 & 0 & 0 & 0 & 0 & 0 & \gamma_H & -\alpha_H & 0 & 0 \\
0 & 0 & 0 & 0 & 0 & 0 & 0 & 0 & -(\gamma_E + b_E) & 0 \\
0 & 0 & 0 & 0 & 0 & 0 & 0 & 0 & \gamma_E & -(b_E + \alpha_E)
\end{pmatrix}$$

The NGM is obtained by multiplying the NGM with large domain by an auxiliary matrix E, as defined in (Diekmann et al., 2010). The eigenvalues and eigenvectors of the NGM are calculated numerically using R 4.2.3.

Detrending of the time series

We calculated all resilience indicators using the rolling window method for time series as described in Dakos et al. (38). For this method, we need to detrend the data to avoid spurious increase of the indicators due to their trend. We used a Gaussian kernel with the optimal bandwidth according to (Bowman & Azzalini 1997), as provided in the package generic_ews in Matlab (https://git.wur.nl/sparcs/generic_ews-for-matlab).
The optimal bandwidth *h* is calculated using

$$hx = \frac{median(\,|x - medx|\,)}{0.6745}\;\frac{\frac{4}{3}}{n}^{0.2}$$

$$hy = \frac{median(\,|y - medy|\,)}{0.6745}\;\frac{\frac{4}{3}}{n}^{0.2}$$

$$h = \sqrt{hy\ \cdot hx}$$

with *x* the time and *y* the time series, and *medx* and *medy* the median of *x* and *y* respectively.

Multivariate Factor Analysis (MAF)

The main essence of Multivariate Factor Analysis (MAF) is to detect the direction of highest autocorrelation, similarly to a PCA with variance (Weinans et al., 2019, 2021).

To find the direction of highest autocorrelation, we proceed as follows:

1. The dataset is transformed to ensure it has an identity matrix as the covariance matrix, using an SDS transform (Haugen et al., 2015). X_{SDS} is the resulting dataset.

2. The first difference of X_{SDS}, $[X_{SDS}(t)-X_{SDS}(t+1)]$, is calculated
3. The eigenvector V and eigenvalues E of the covariance matrix of the first difference $[X_{SDS}(t)-X_{SDS}(t+1)]$ are calculated
4. The dataset is projected in the direction of highest autocorrelation by multiplying by V

It is important to note that MAF is inverted compared to a PCA: the minimum eigen value of the MAF corresponds to the maximum eigen value for a PCA.

Multivariate indicators of resilience for different R_0

To test the behaviour of our indicators for different values of R_0, we generated time series under different fixed R_0 values under the critical threshold (i.e. <1), and analyse each time series separately. We call these data "fixed R_0 runs". Each of these time series was simulated over 70 weeks, with a weekly resolution, for 100 values of R_0 between 0.2 and 1. For each value of R_0, 100 stochastic repetitions were simulated. The value of the different indicators was then calculated in each repetition for each value of R_0, and the median and 95% interval were determined for each value of R_0. This procedure was repeated for each monitoring scenario and each indicator (univariate and multivariate).

The slowing down observed in the perturbation recovery experiments (Figure 2) was reflected by an increase in resilience indicators in our simulated fixed time series (Figure A to C). Especially variance-based indicators such as mean variance, max variance and PCA variance displayed a strong increase as R_0 increased. Autocorrelation based indicators such as MAF autocorrelation, mean autocorrelation and max autocorrelation displayed a weaker increase. Additionally, autocorrelation-based indicators sometimes were high for low values of R_0. Due to the scarcity of cases far from the epidemic threshold, the time series contained long stretches of consecutive zeros, resulting in a highly variable autocorrelation, but these zeros obviously did not reflect any measure of the recovery speed of the system.

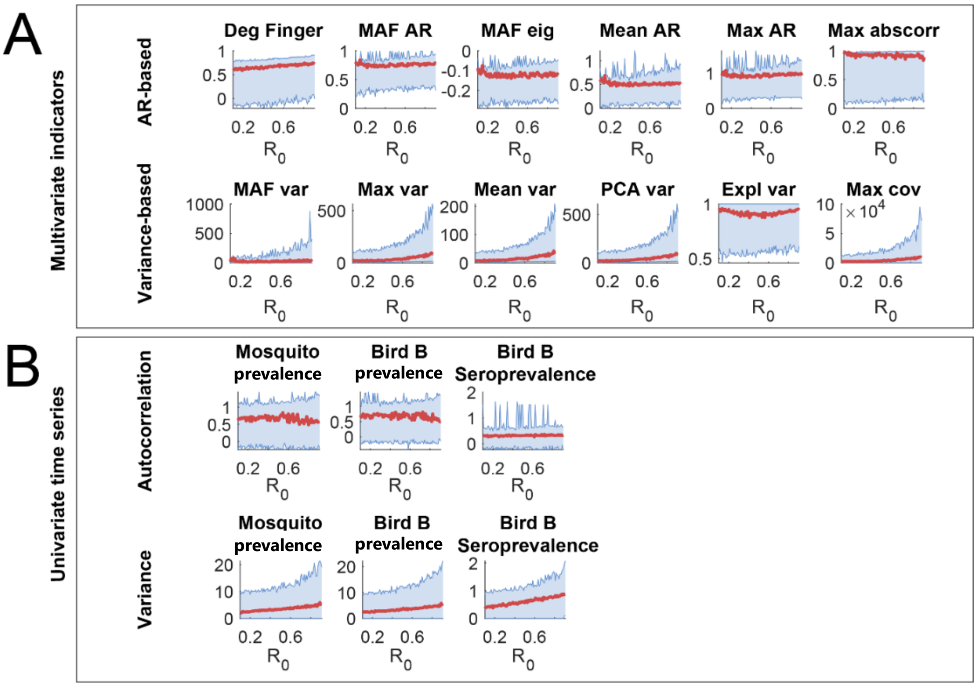

Figure A. Indicators of resilience, calculated from separate time series generated for each level of R_0 (i.e. fixed R_0 runs) following the hidden reservoir monitoring scenario. The red line indicates the median estimation over all the repetitions, and the blue lines are the 2.5 and 97.5 percentiles. (A) Multivariate

indicators of resilience, calculated in fixed runs of the multivariate time series following the hidden scenario. (B) Univariate indicators of resilience calculated in fixed runs of univariate time series contained in the hidden reservoir scenario.

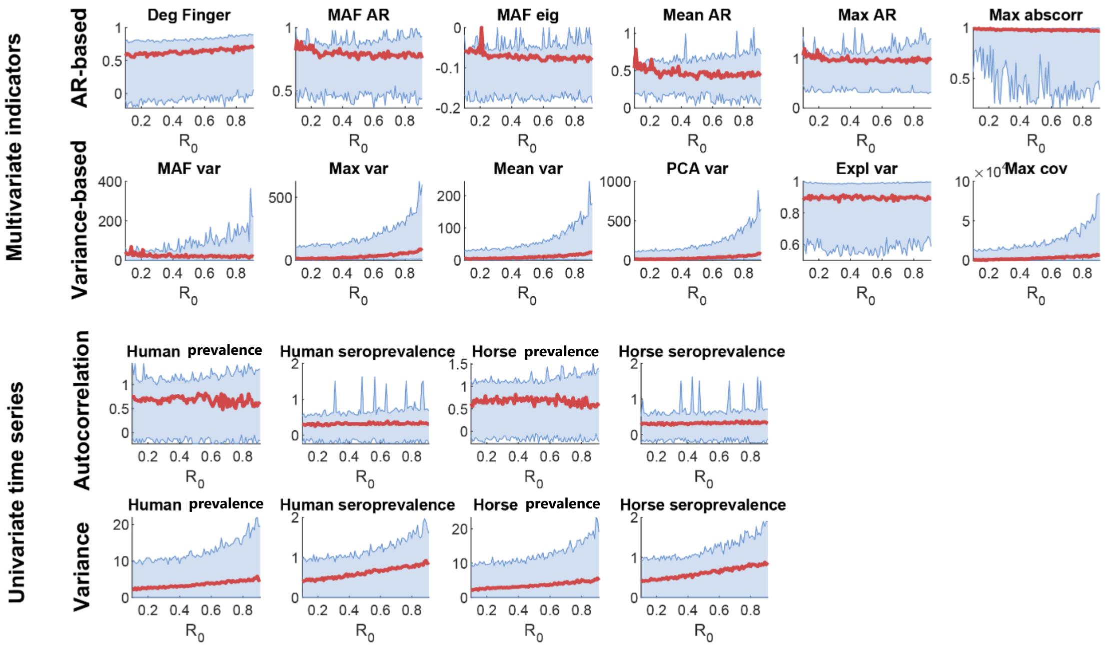

Figure B. Multivariate indicators of resilience, calculated from separate time series of the anthro-equine scenario generated for each level of R_0 (i.e. fixed R_0 runs, see Methods). The solid line indicates the median estimation over all the repetitions, and the dotted lines are the 2.5 and 97.5 percentiles.

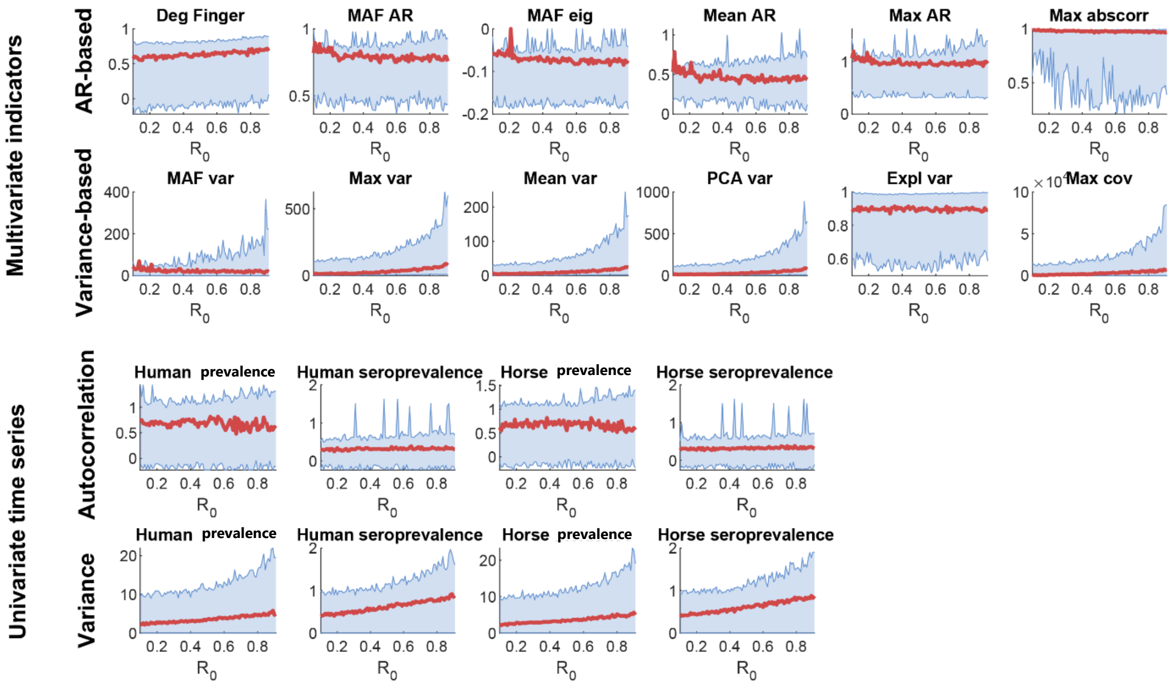

Figure C. Multivariate indicators of resilience, calculated from separate time series of the wildlife scenario generated for each level of R_0 (i.e. fixed R_0 runs, see Methods). The solid line indicates the median estimation over all the repetitions, and the dotted lines are the 2.5 and 97.5 percentiles.

Down sampling of the data for all the indicators and monitoring scenarios

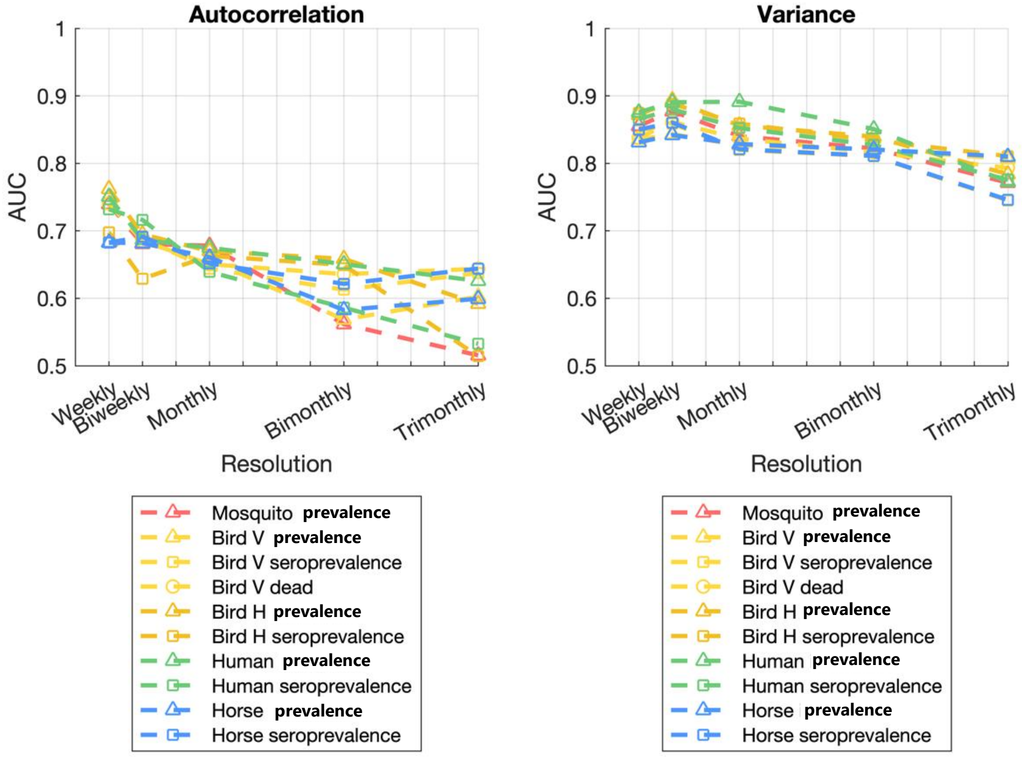

Figure D. Prediction performance all univariate time series for both autocorrelation and variance, depending on the resolution of the data.

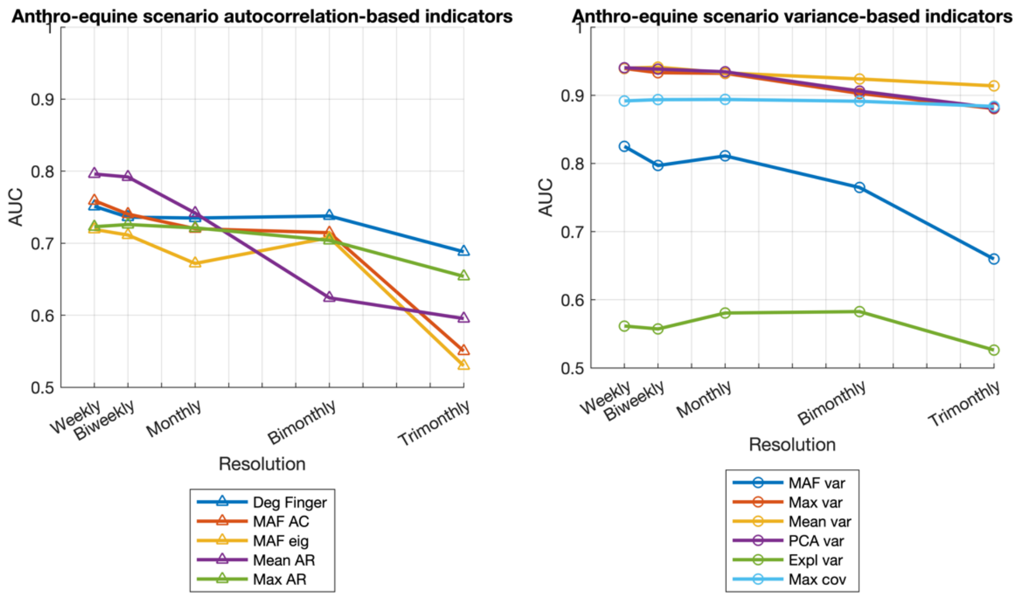

Figure E. Prediction performance for the anthro-equine scenario for all autocorrelation and variance-based indicators, depending on the resolution of the data.

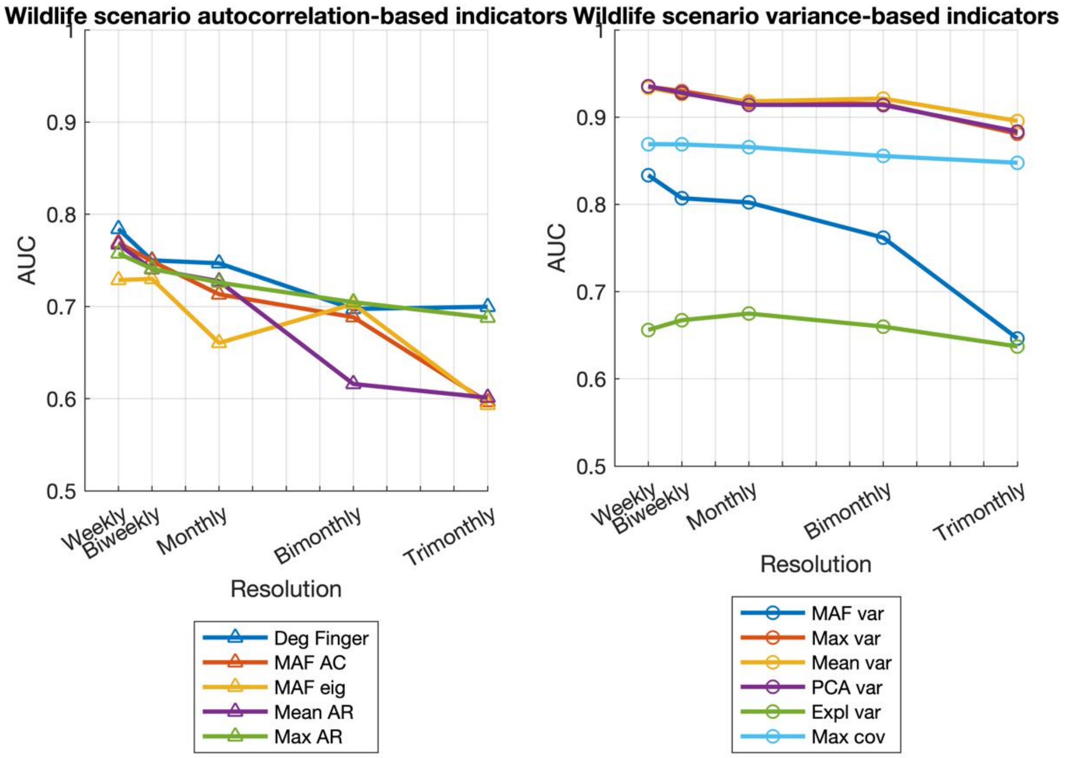

Figure F. Prediction performance for the wildlife scenario for all autocorrelation and variance-based indicators, depending on the resolution of the data.

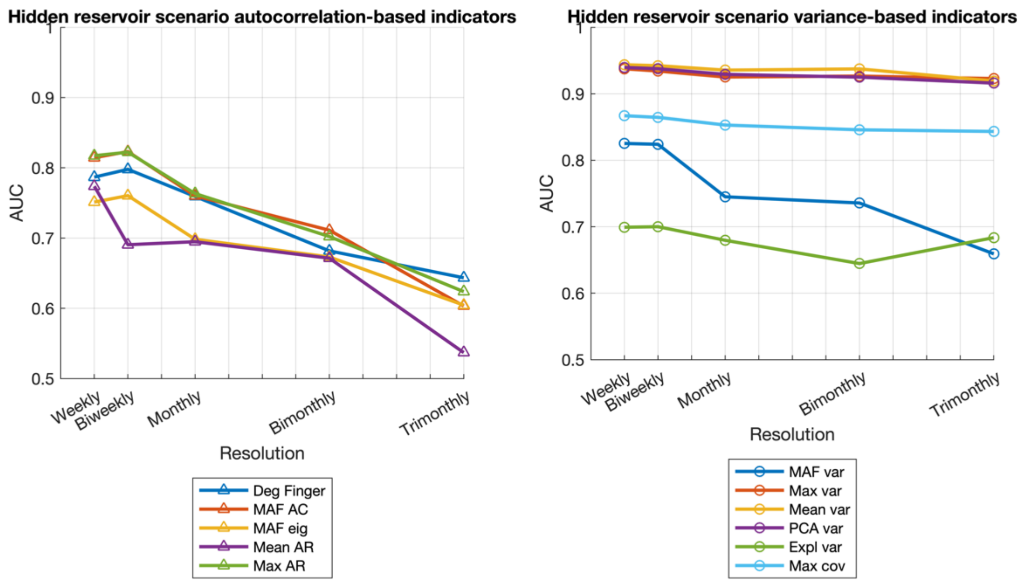

Figure G. Prediction performance for the hidden reservoir scenario for all autocorrelation and variance-based indicators, depending on the resolution of the data.

Reducing the observation probability for all the indicators and monitoring scenarios

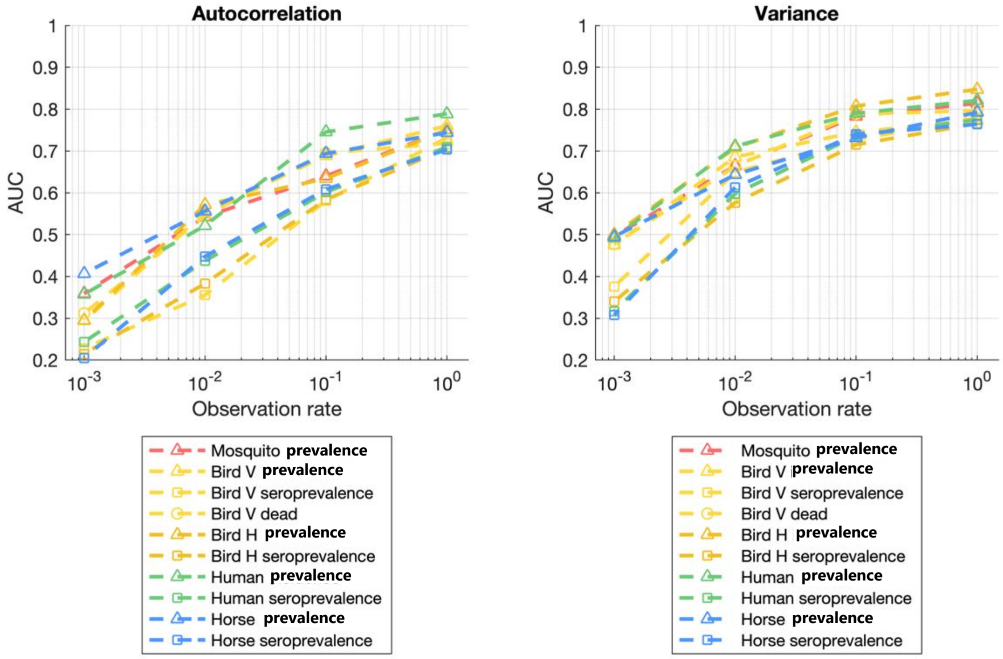

Figure H. Prediction performance all univariate time series for both autocorrelation and variance, depending on the observation probability.

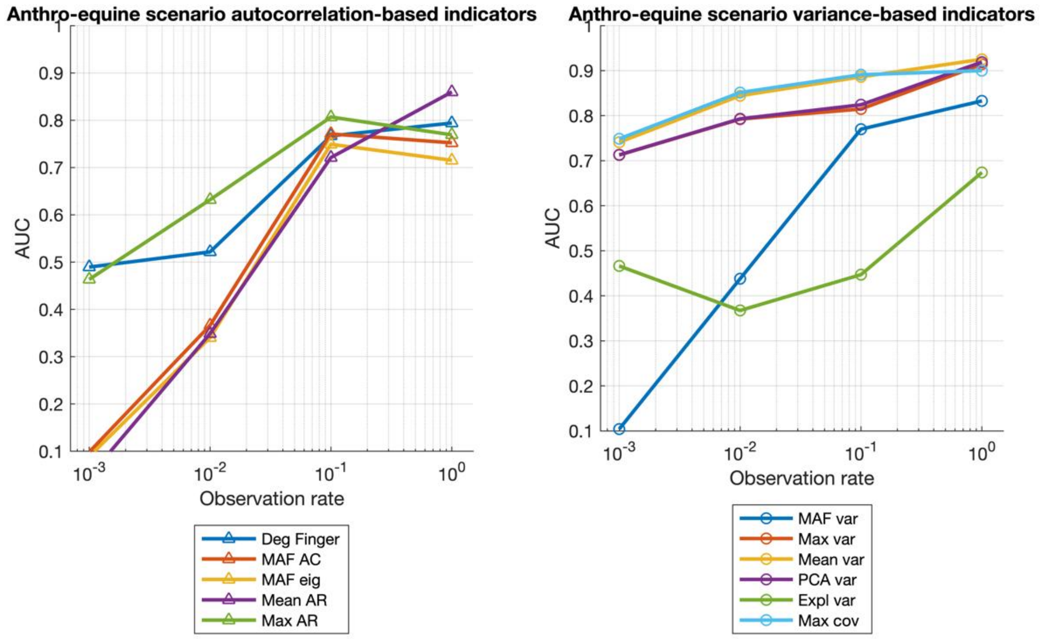

Figure I. Prediction performance for the anthro-equine scenario for all autocorrelation and variance-based indicators, depending on the observation probability.

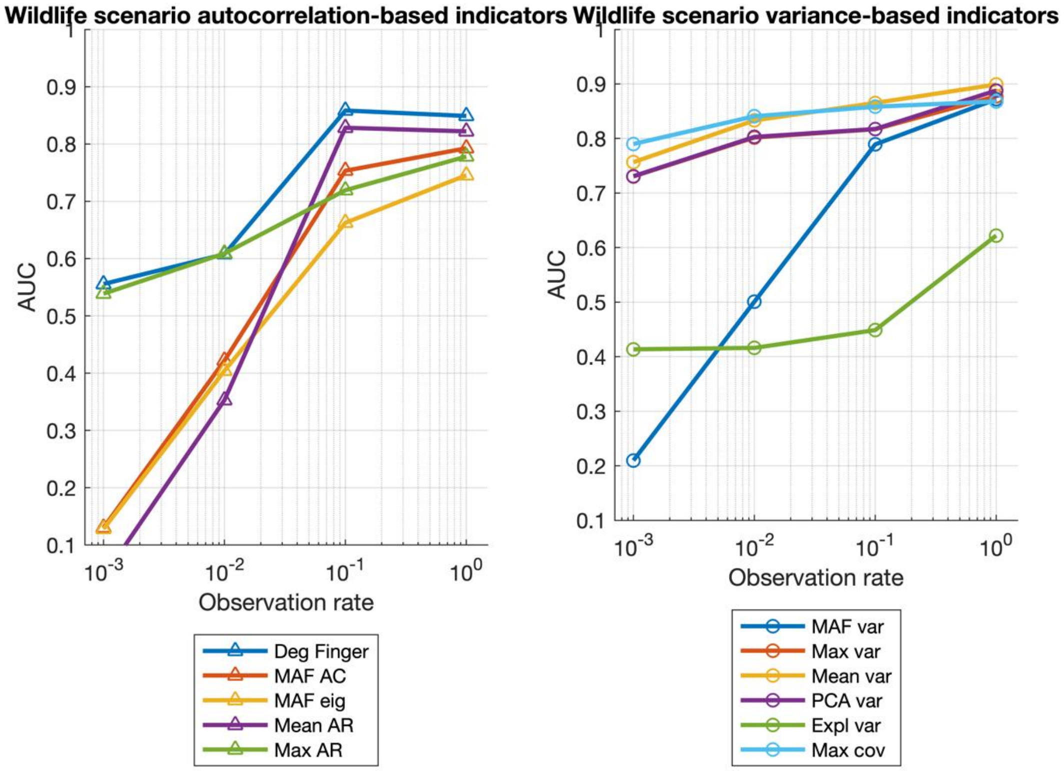

Figure J. Prediction performance for the wildlife scenario for all autocorrelation and variance-based indicators, depending on the observation probability.

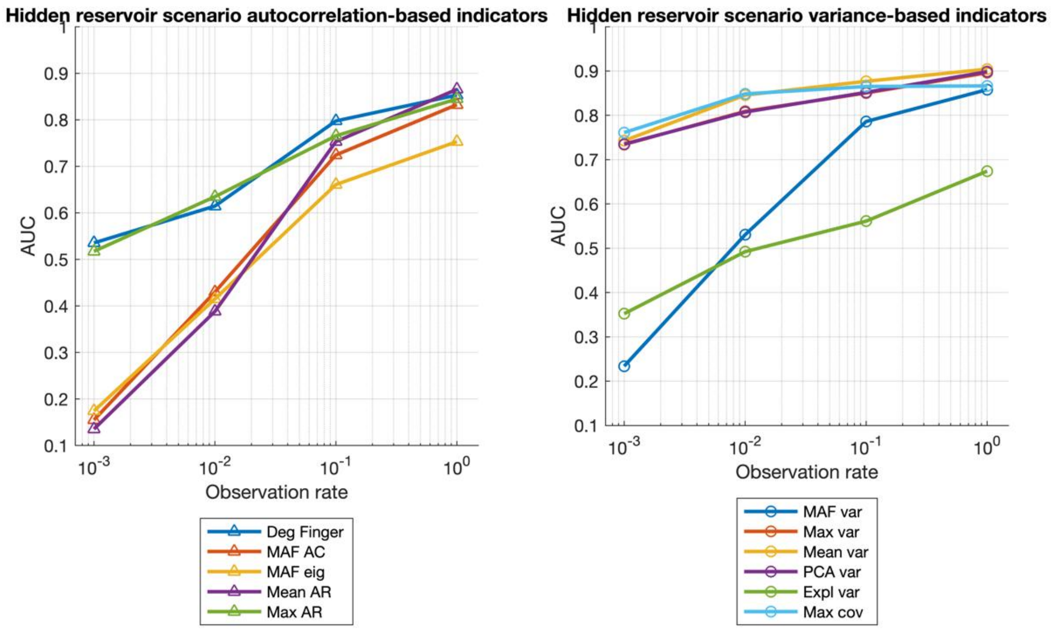

Figure K. Prediction performance for the hidden reservoir scenario for all autocorrelation and variance-based indicators, depending on the observation probability.

Change in mosquito feeding preference for all the indicators and monitoring scenarios

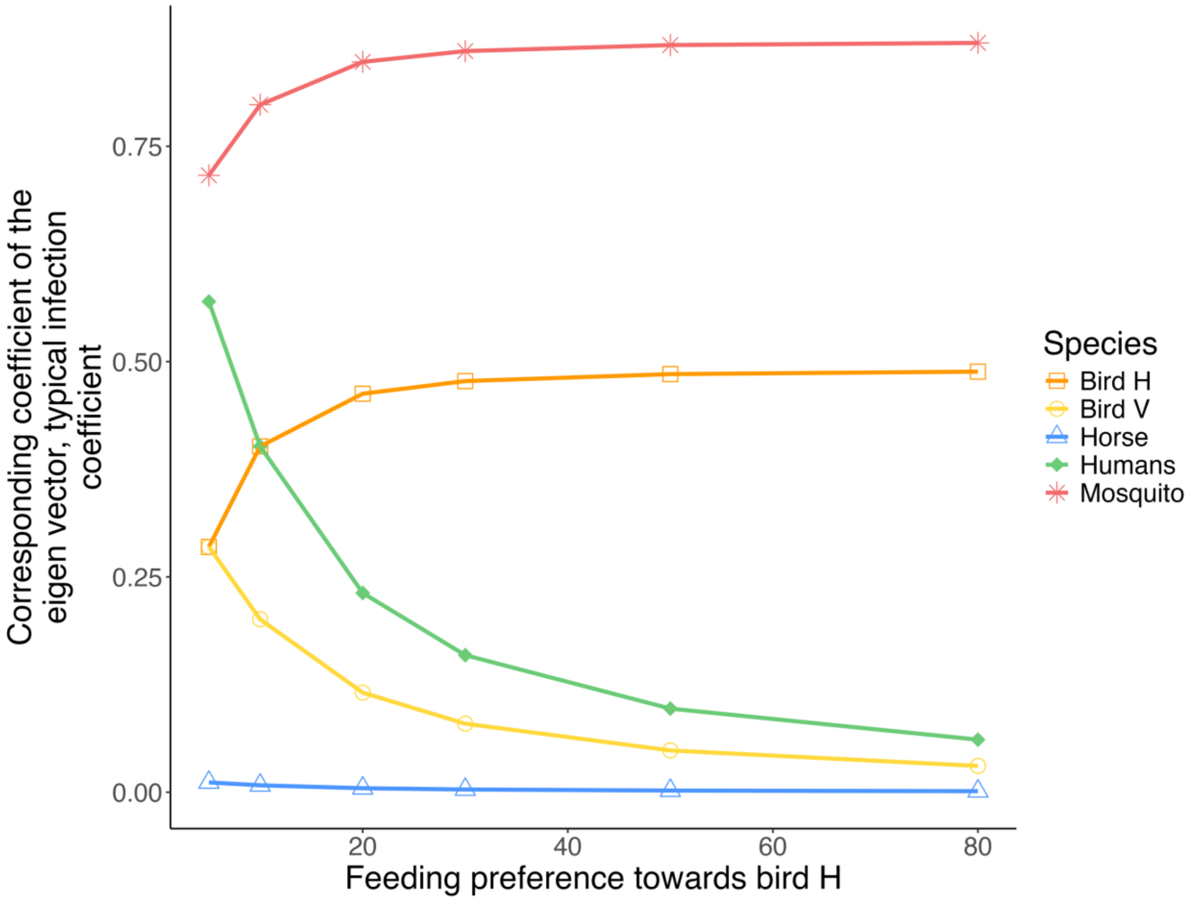

Figure L. Relation between the feeding preference towards bird H and the *typical infection coefficient*, i.e. the corresponding coefficient of the eigenvector associated with the dominant eigenvalue for each species in the model.

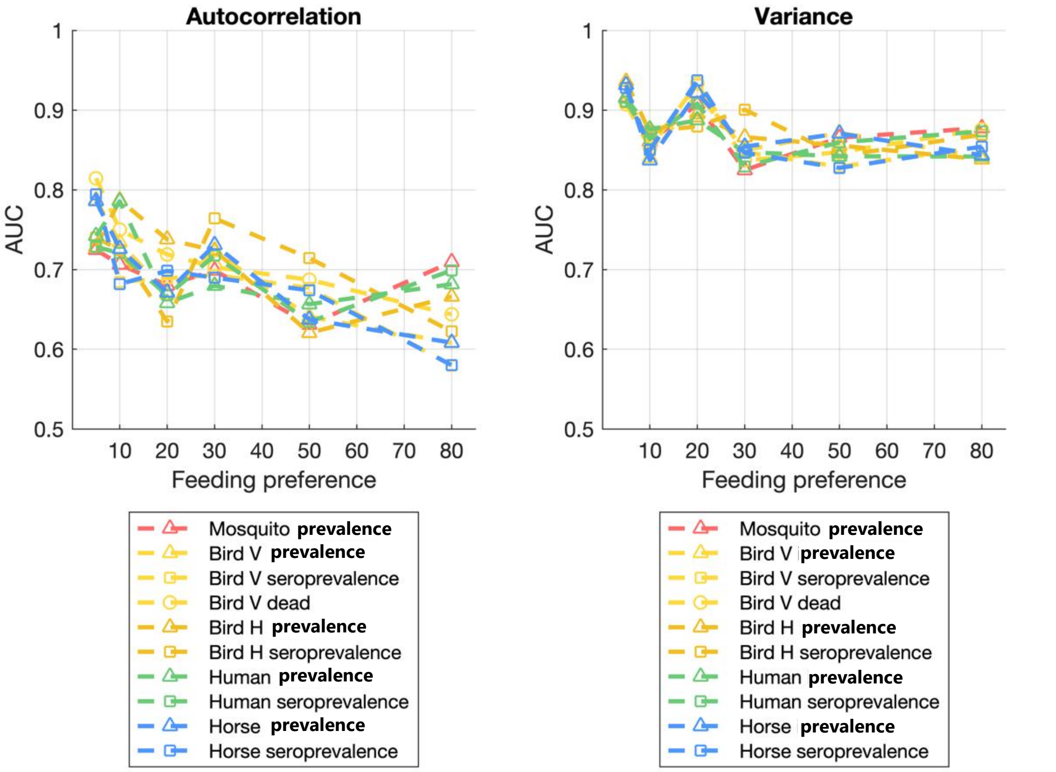

Figure M. Prediction performance all univariate time series for both autocorrelation and variance, depending on the feeding preference towards bird H.

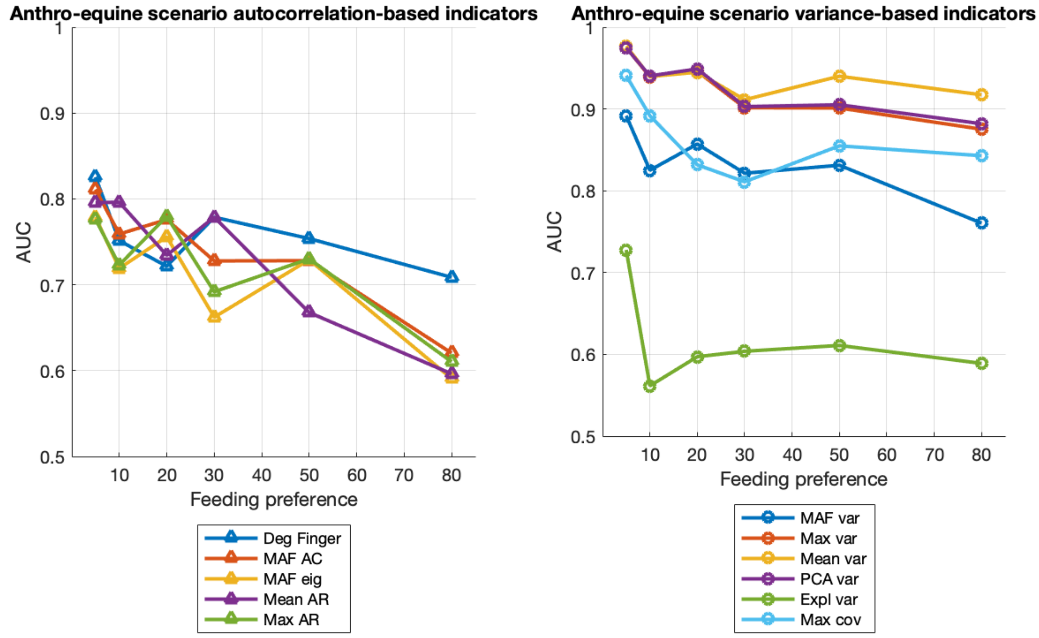

Figure N. Prediction performance for the anthro-equine scenario for all autocorrelation and variance-based indicators, depending on the feeding preference towards bird H.

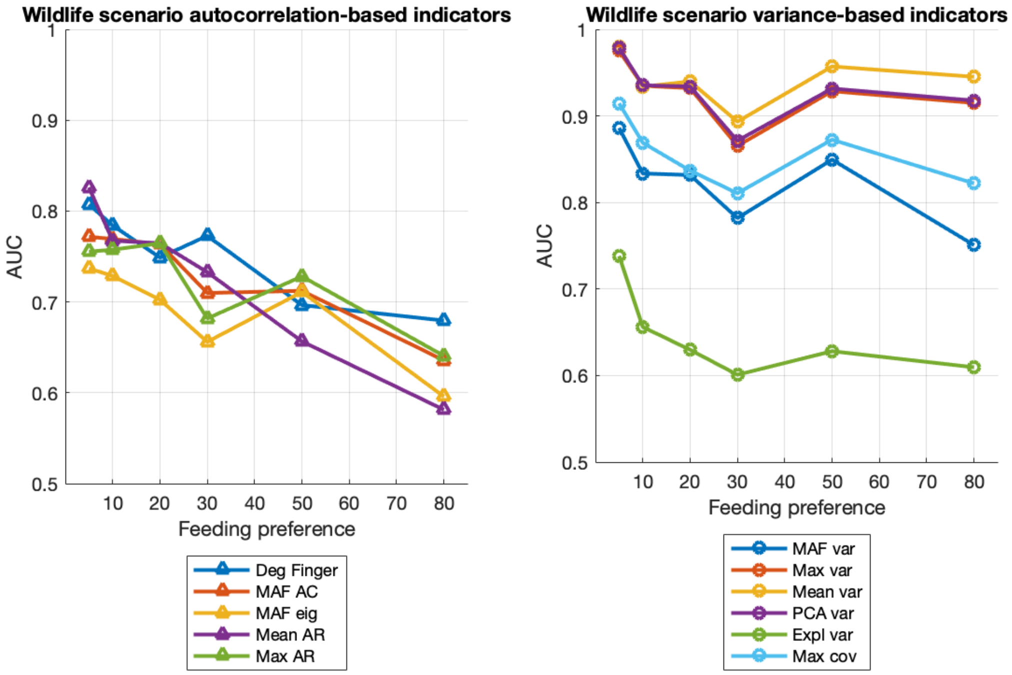

Figure O. Prediction performance for the wildlife scenario for all autocorrelation and variance-based indicators, depending on the feeding preference towards bird H.

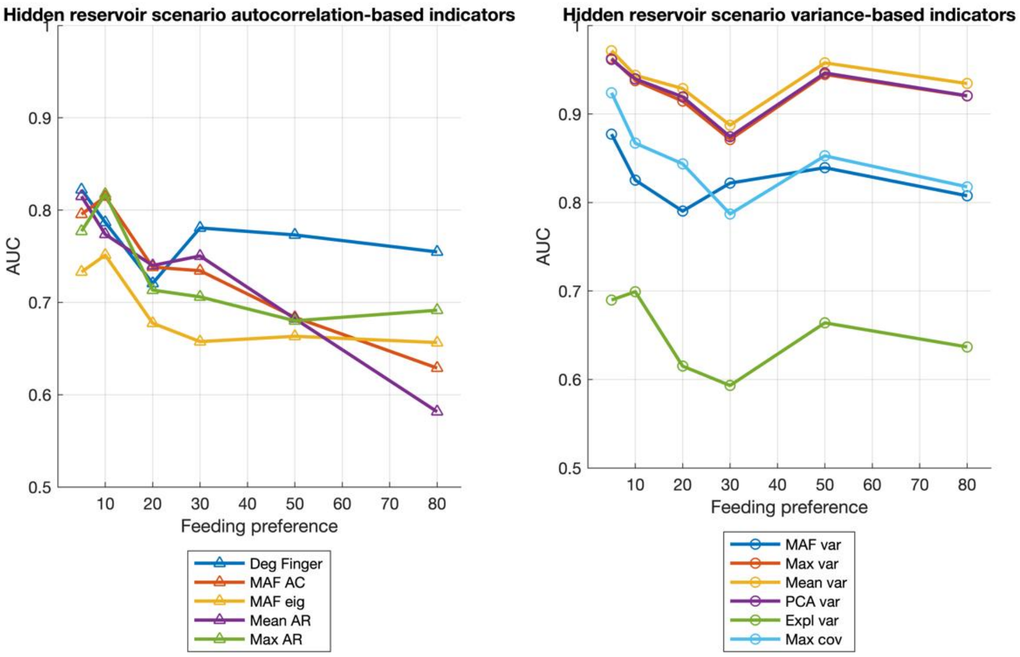

Figure P. Prediction performance for the hidden reservoir scenario for all autocorrelation and variance-based indicators, depending on the feeding preference towards bird H.

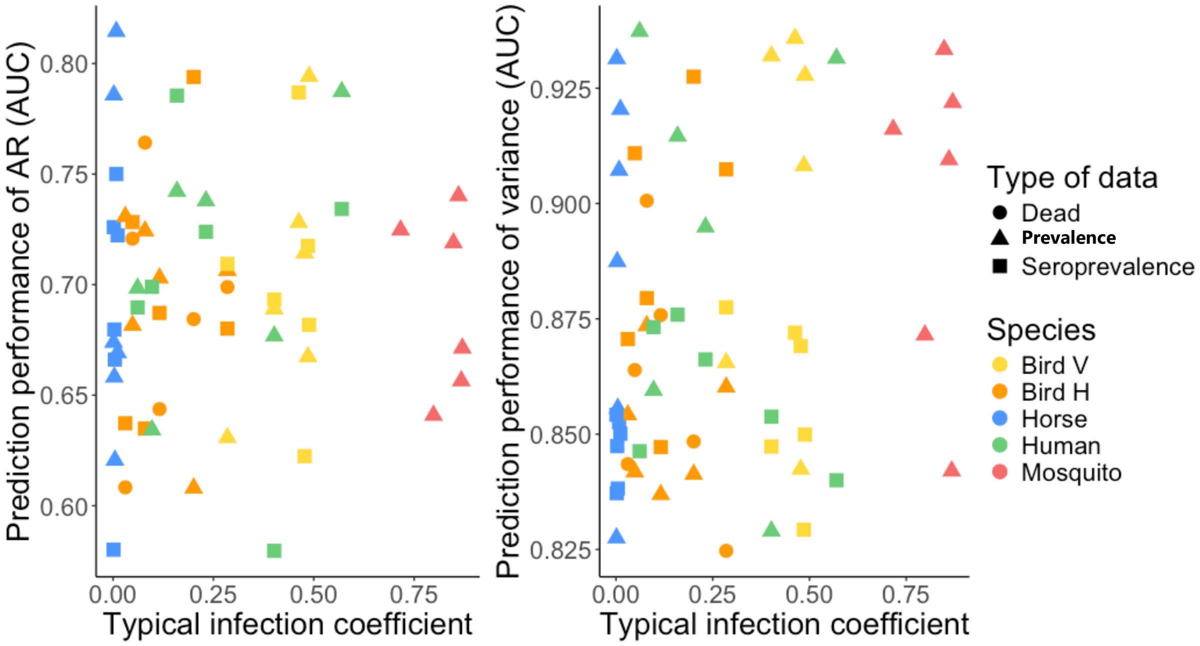

Figure Q. Prediction performance of univariate time series depending on the *typical infection coefficient*, i.e. the corresponding coefficient of the eigenvector associated with the dominant eigenvalue for each species in the model.

Robustness of the results of different feeding preference for lower resolution and observation probability

The results of Figure D (reducing the resolution), and Figure H (reducing the observation probability) are reproduced for the most extreme feeding preference towards bird H (p_{BH}=80) to test the robustness of the results of Figure M and verify that no variability in the prediction performance of the different species arises.

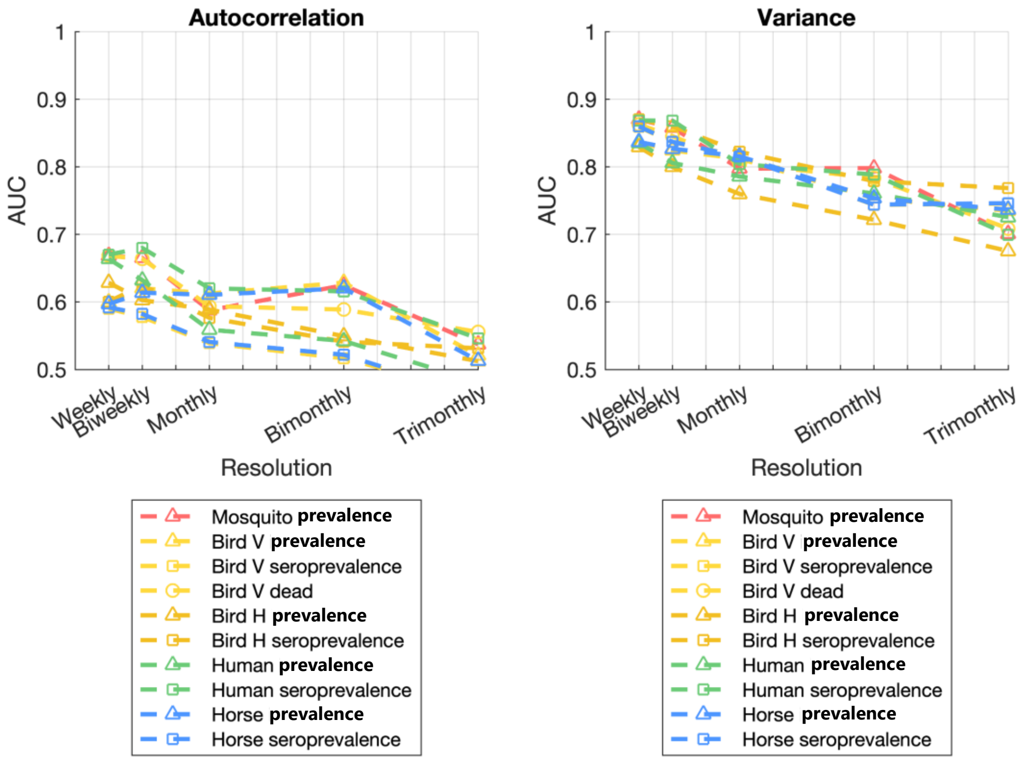

Figure R: Prediction performance all univariate time series for both autocorrelation and variance, depending on the resolution of the data, for a strong feeding preference towards bird H (p_{BH}=80).

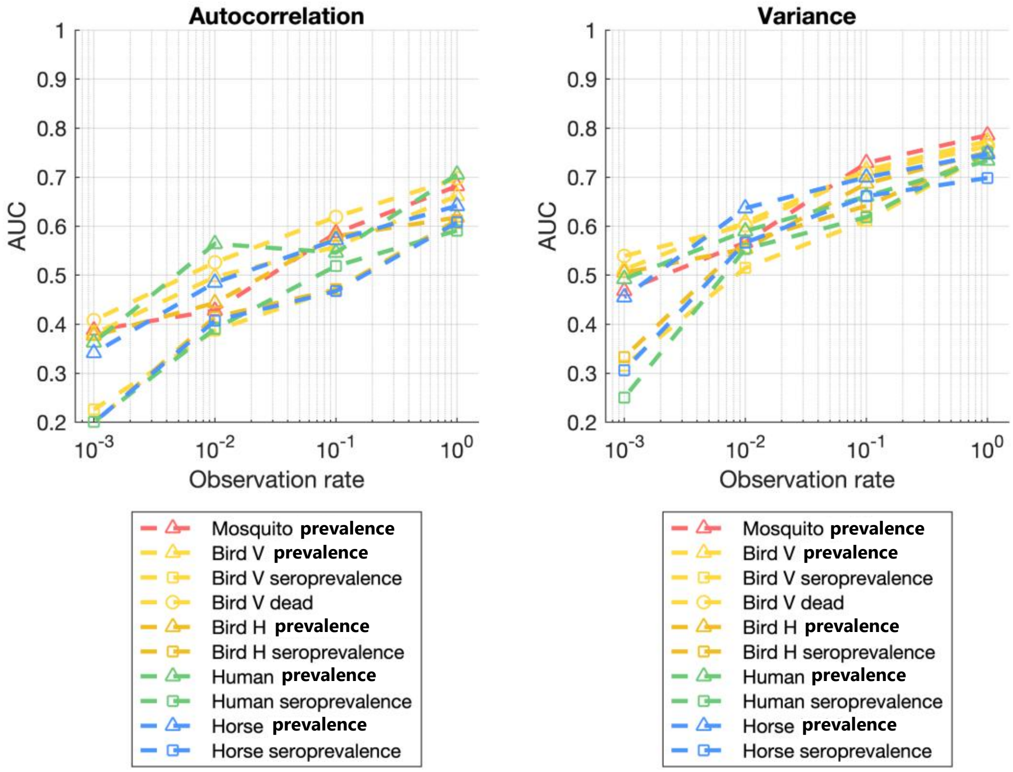

Figure S: Prediction performance all univariate time series for both autocorrelation and variance, depending on the resolution of the data, for a strong feeding preference towards bird H (p_{BH}=80).

Change in bird relative abundances as a sensitivity analysis

To make sure that the effect of *the typical infection coefficient* on the prediction performance of a given species is not singular to the feeding preference coefficient, we reproduced the same analyses by varying the relative abundance of bird H compared to bird V and keeping the feeding preference coefficient at the default value. We generated time series of the model with 4 values for the relative abundance of bird species H. In the default emergence time series, bird H has the same abundance as bird species V (relative abundance r_A = 1). Additionally, we generated time series with r_A=0.75, 1.25, and 1.5. Similarly to the analyses performed by varying the feeding preference coefficient, for each simulation, we adapted the range of values for the biting rate to keep a range of R_0 going from 0.7 to 1 for the emergence time series, and R_0=0.8 for the stable time series.

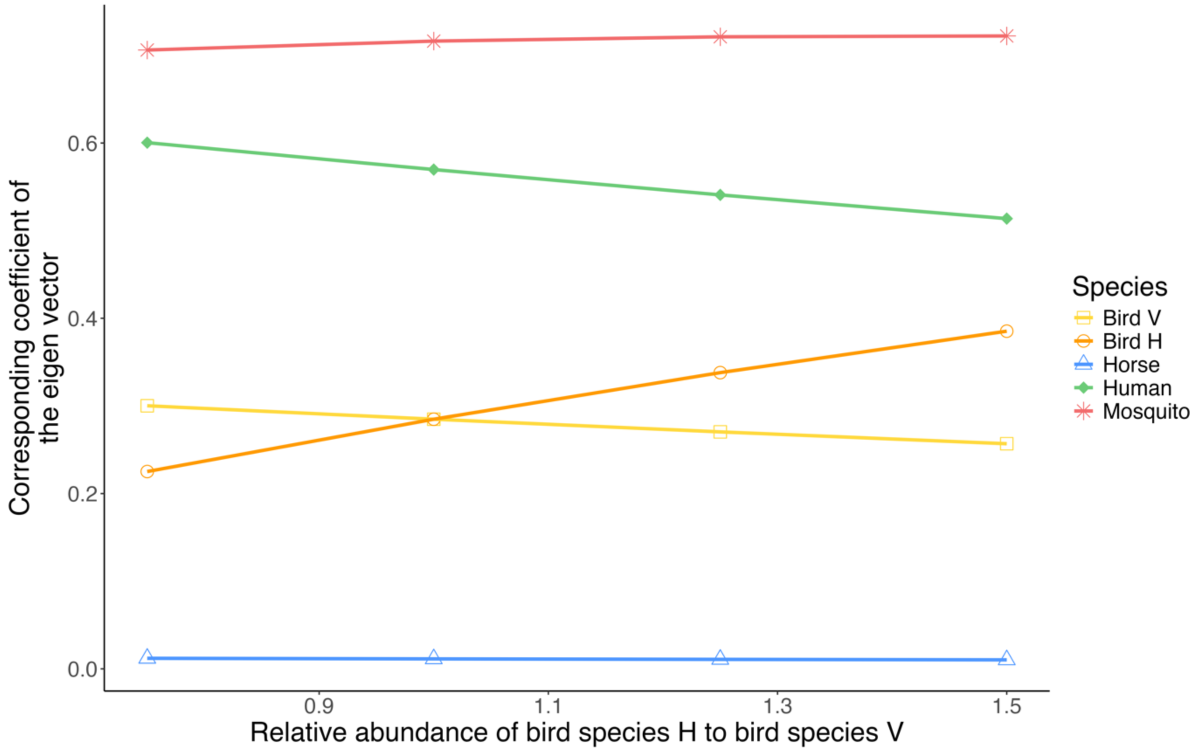

Figure T. Relation between the relative abundance of bird H and the *typical infection coefficient*, i.e. the corresponding coefficient of the eigenvector associated with the dominant eigenvalue for each species in the model.

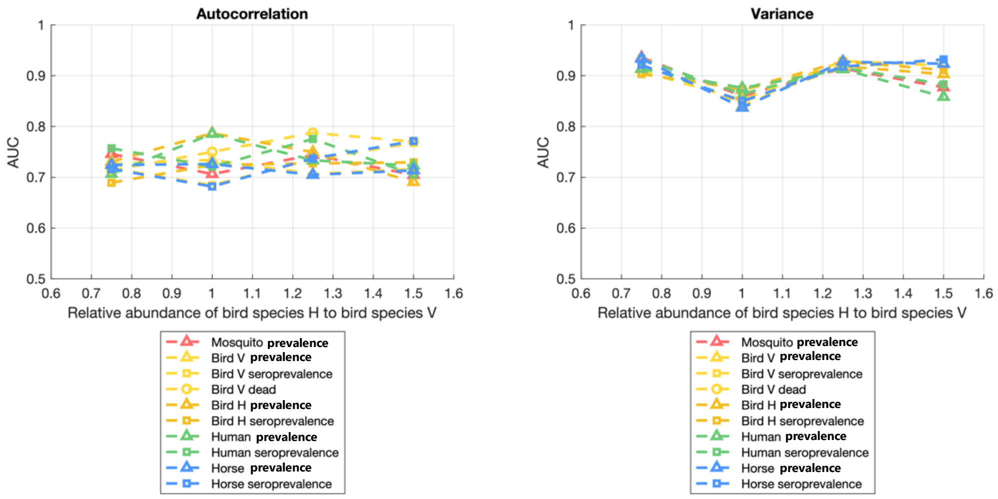

Figure U. Prediction performance all univariate time series for both autocorrelation and variance, depending on the relative abundance of bird H.

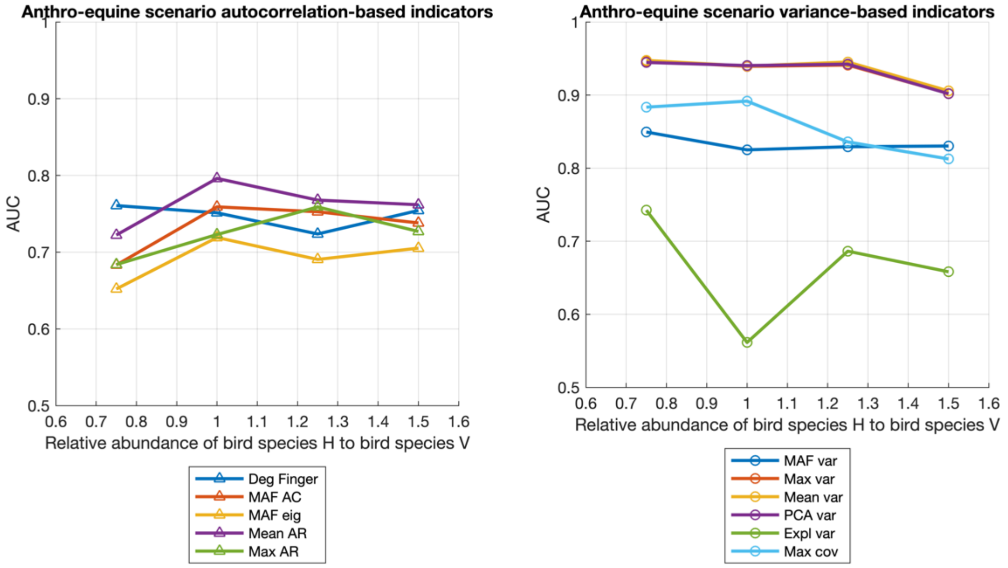

Figure V. Prediction performance for the anthro-equine scenario for all autocorrelation and variance-based indicators, depending on the relative abundance of bird H.

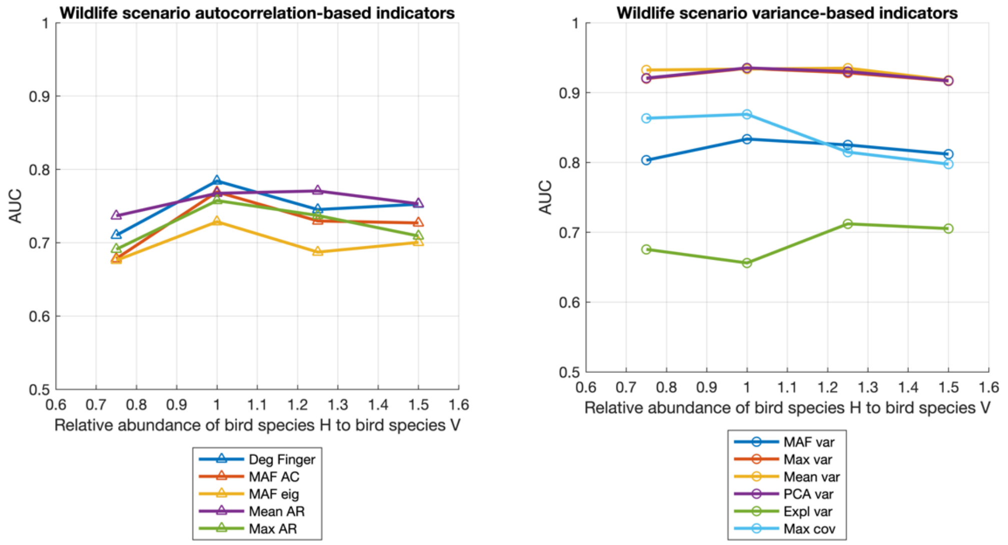

Figure W. Prediction performance for the wildlife scenario for all autocorrelation and variance-based indicators, depending on the relative abundance of bird H.

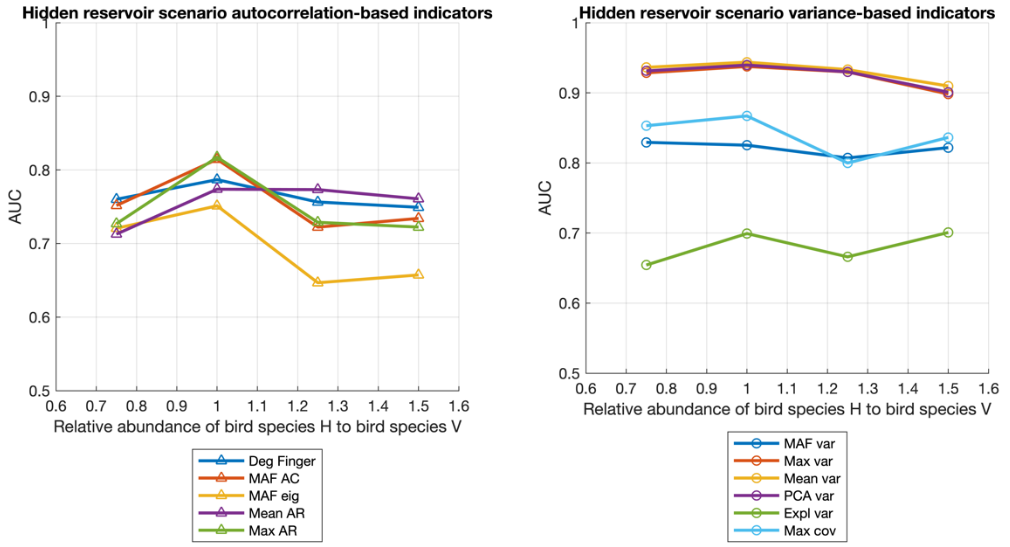

Figure X. Prediction performance for the hidden reservoir scenario for all autocorrelation and variance-based indicators, depending on the relative abundance of bird H.

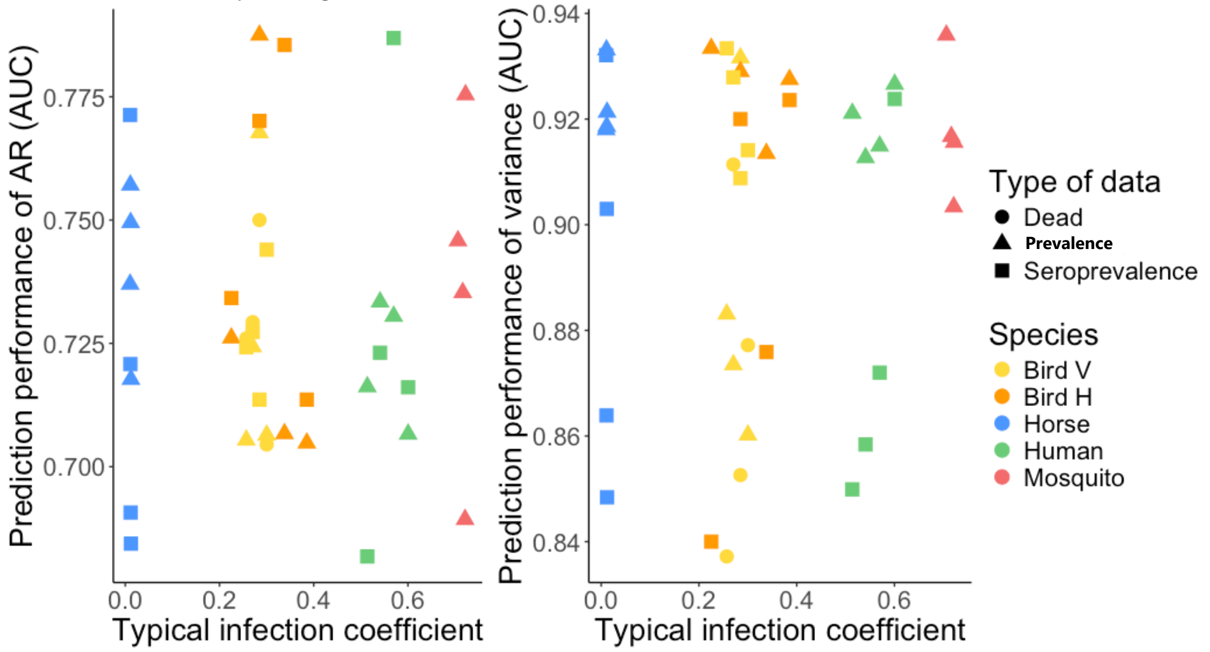

Figure Y. Prediction performance of univariate time series depending on the *typical infection coefficient*, i.e. the corresponding coefficient of the eigenvector associated with the dominant eigenvalue for each species in the model, calculated by varying the relative abundance of bird species H.

Lead time of prediction

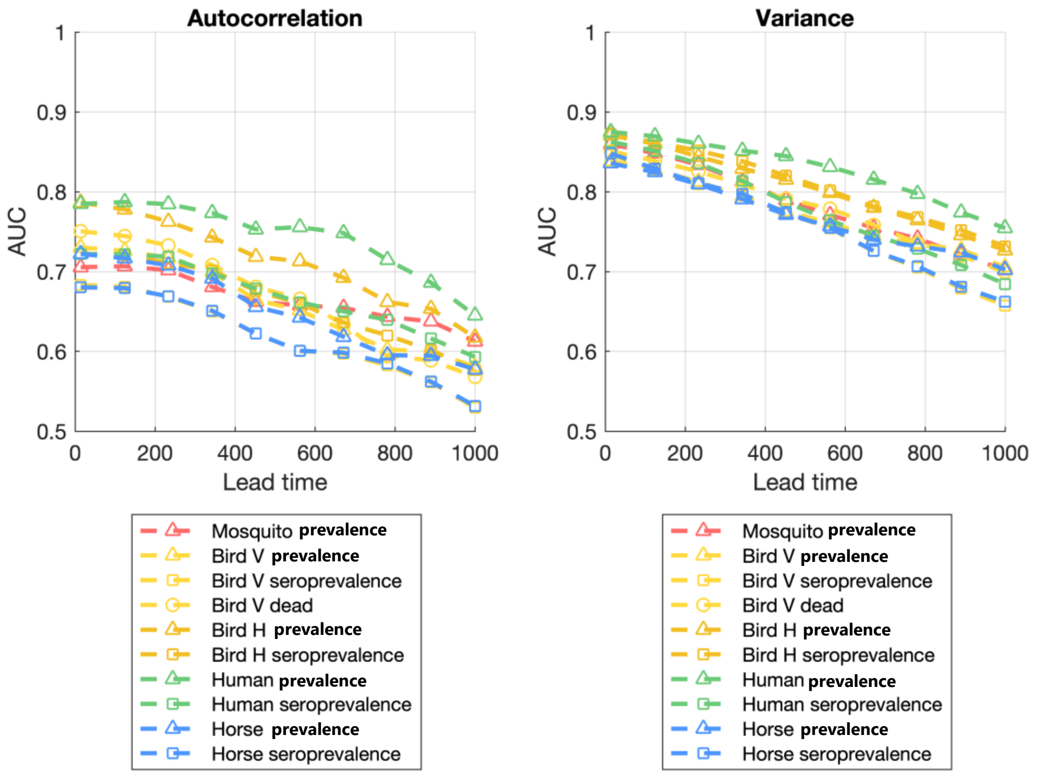

Figure Z. Prediction performance all univariate time series for both autocorrelation and variance, for different lead times (days).

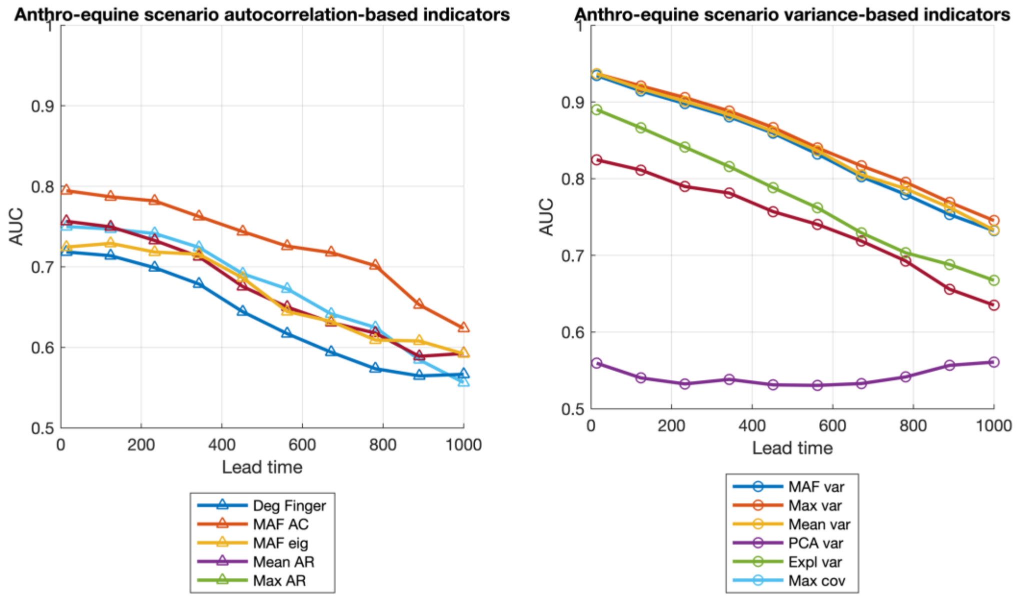

Figure AA. Prediction performance for the Anthro-equine scenario for all autocorrelation and variance-based indicators, for different lead times (days).

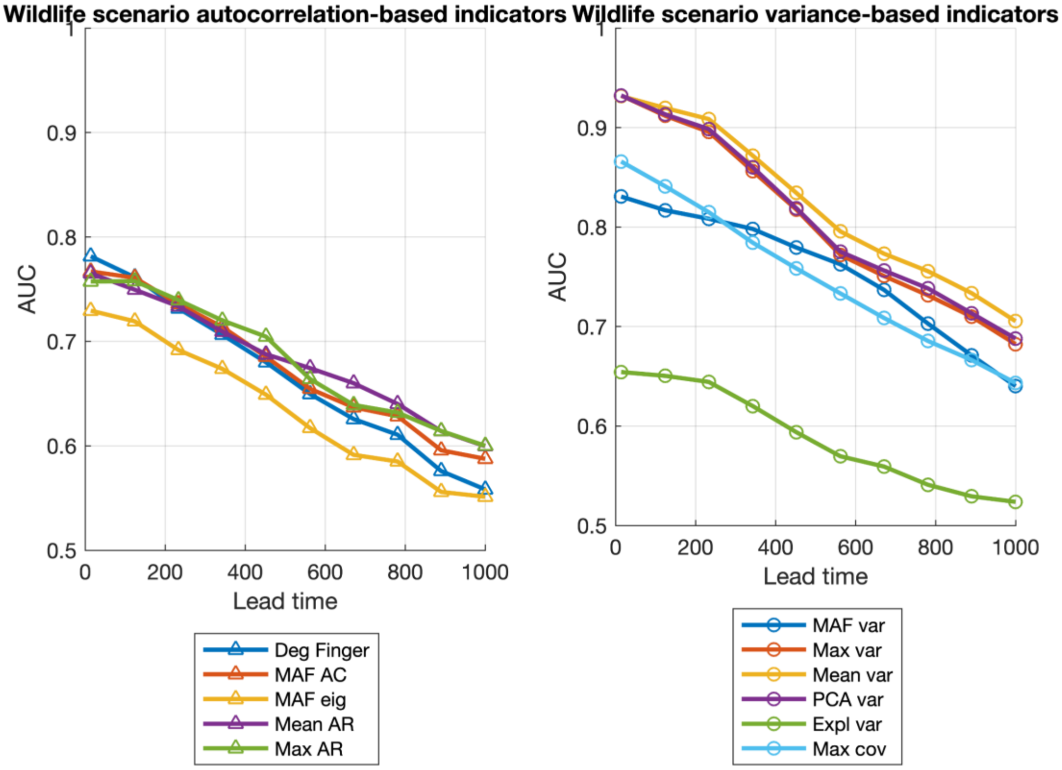

Figure AB. Prediction performance for the Wildlife scenario for all autocorrelation and variance-based indicators, for different lead times (days).

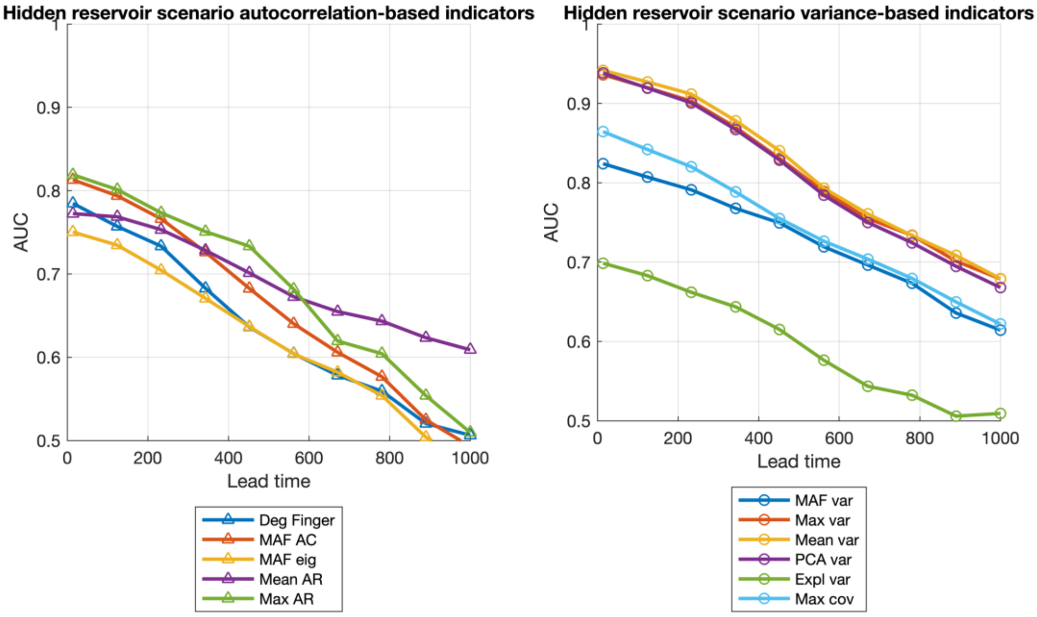

Figure AC. Prediction performance for the hidden reservoir scenario for all autocorrelation and variance-based indicators, for different lead times (days).

Additional analyses to explain the changes in AUC

Surprisingly, the prediction performance of the different multivariate monitoring scenarios, as well as univariate time series, remained stable regardless of how much a species is involved in the transmission cycle. We investigated additional properties of the time series that could drive the prediction performance of resilience indicators, we could not find a satisfactory explanation (Figure AD).

These analyses were run similarly to the analyses presented in the manuscript for Figure 5B: the AUC of the univariate indicators was calculated for the different univariate time series generated when varying the biting preference of the mosquitoes towards the hidden bird reservoir. However, here we investigated the effect of intrinsic properties of the time series on the AUC: the number of zeros in the time series (Figure AD A and B), the amplitude of the time series, calculated as *maximum y – minimum y* (Figure AD C and D), the maximum value in the time series (Figure AD E and F) and the sum of all the values in the time series (Figure AD G and H).

For instance, a species with a relatively low involvement in the transmission cycle would exhibit a high number of zeros due to the rare observation of cases (Figure AD A and B), whereas a species with a high involvement will exhibit a high maximum in the time series (Figure AD E and F) and a larger amplitude (Figure AD C and D). However, none of these processes seemed to influence the prediction performance quantified using the AUC. Further analyses are required to understand what really drives how informative a data source is.

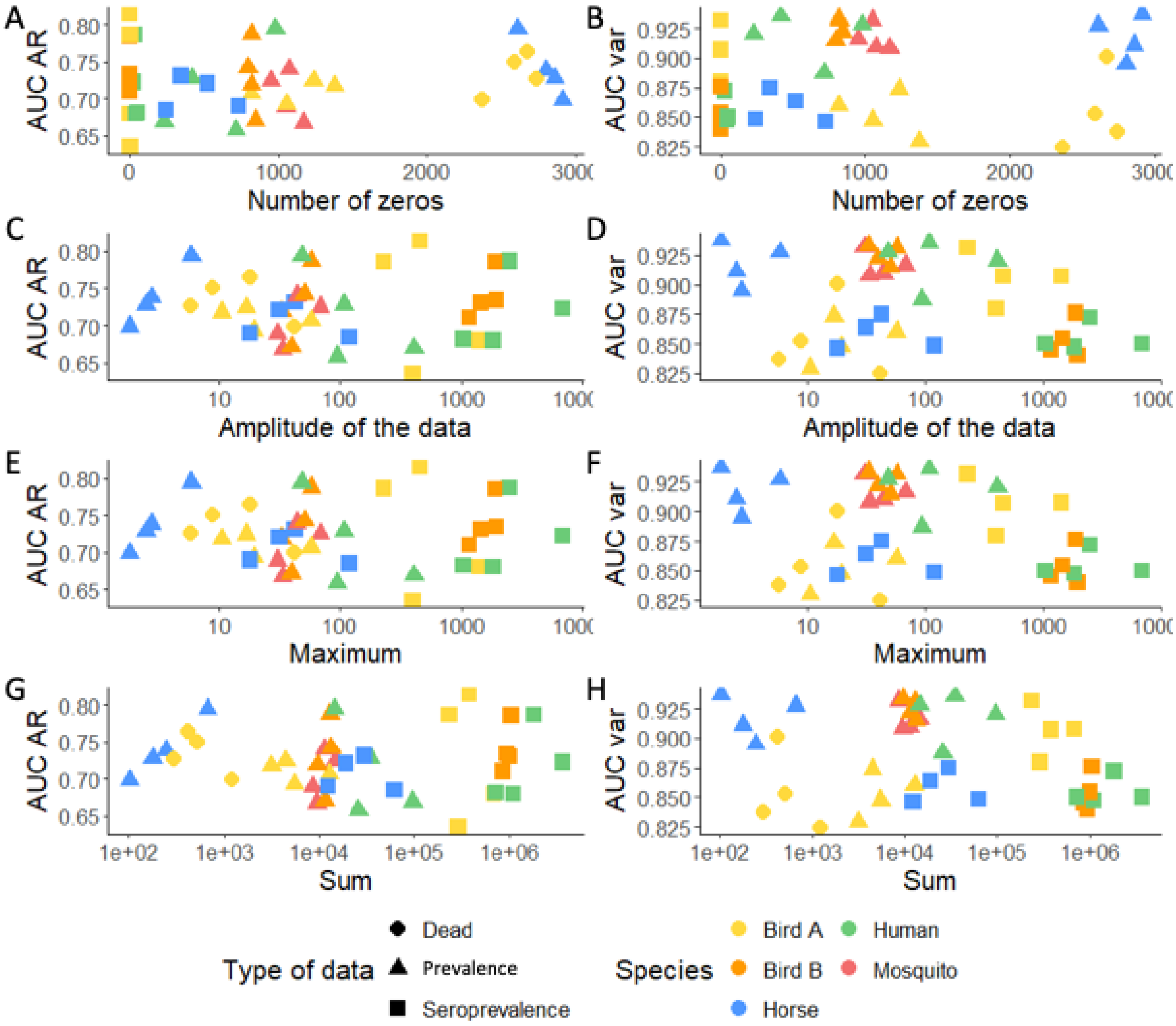

Figure AD: Performance of univariate resilience indicators for different properties of the time series. Autocorrelation (A, C, E, F) and variance (B, D, F, H) are evaluated using the AUC. Effect of the number of zeros (A, B), amplitude of the data defined as *max-min* (C, D), maximum in the time series (E, F) and total sum of the time series (G, H) on the performance of the indicators.
