# Supplementary material for: Multivariate resilience indicators to anticipate vector-borne disease outbreaks: A West Nile virus case-study
Source: PLoS Comput Biol. 2025 Oct 13;21(10):e1012703. doi: 10.1371/journal.pcbi.1012703 (PMC12539738; doi:10.1371/journal.pcbi.1012703)
Supplement: Attachment — Submitted filename: Rebuttal letter.pdf [file pcbi.1012703.s004.pdf]

Juliette Paireau
Academic Editor
PLOS Computational Biology

Roger Kouyos
Section Editor
PLOS Computational Biology

10-05-2025

Rebuttal letter of manuscript titled 'Multivariate resilience indicators to anticipate vector-borne disease outbreaks: a West Nile virus case-study'

Dear Editors,

We thank the reviewers for their generous comments on the previous version of the manuscript. We have now addressed their concerns in a revised version. In particular, we have clarified the methodology, specifically for the calculation of resilience indicators and performance assessment.

With kind regards,

Clara Delecroix,
On behalf of all authors.

Editor Comments:

The Authors are expected to address all the points raised by the Reviewers. In particular, the authors should give more details on the definition of resilience indicators (reviewers #1 and #3) and clarify the methodology used for performance assessment (reviewer #2). In addition, the code should be made available in the provided Github repository.

Thank you for your comments. We respond to the reviewers in the rest of this letter. The code has been made available in the GitHub repository

https://github.com/cla-delec/Multivariate_resilience_indicators.

Reviewers Comments:

Reviewer #1:
The manuscript provides an interesting modelling analysis aiming at evaluating the efficacy of resilience indicators to anticipate a vector borne outbreak, using synthetic West Nile virus epidemiological data. While finding the study well written and of interest, some lacking details and some doubts prevent me from fully appreciating it.

Thank you for reviewing our manuscript. We tried to include additional details accordingly. We respond to your comments one-by-one below.

Major points
1. Throughout the manuscript the term "incidence" is used but I believe you actually mean prevalence. Incidence is defined as the number of new infections per unit of time, whereas prevalence is the fraction of infectious individuals, which is what is typically observed (for instance, analyzing mosquito pools provide an indication of the mosquito prevalence). Compartments MI, BVI, BHI, HI and EI denote the number of infectious individuals at time t, so again to me this is prevalence and not incidence. Please check carefully what you mean and amend the text accordingly.

Thank you for pointing out that mistake in terminology. We indeed meant prevalence, we have amended the manuscript accordingly.

2. Figure 1B. Why is there a sudden decrease in the number of infectious mosquitoes when R_0 becomes greater than 1? This is rather counterintuitive. Plots show a single simulation, right? They are not the average of the 100 simulations, right?

Figure 1B and 1C are indeed a single simulation. Thus, this decrease is due to intrinsic stochasticity in the model. We have now indicated that more clearly in the legend, and we have changed the example to time series without this drop to prevent confusion to the reader.
"*Fig 1. WNV model and simulated data (A) WNV compartmental model used in the analyses. (B) Example of a simulated time series with an upcoming epidemic, simulated by increasing R_0 from 0.7 to 1. The shaded region indicates where R_0 is above 1. (C) Example of a simulated time*

series with no upcoming epidemic, simulated with a stable R_0=0.8. In (B) and (C), the time series represent a single simulation and were aggregated per month to improve the readability of the figure."

3. I am not familiar with resilience indicators hence it is not clear to me what the authors mean and use. I would have expected some equations and more details illustrating what they are and how they are computed but these important points are currently missing. Please add some text, some examples and some equations (you can place them in the Appendix).

Thank you for your comment. We have amended the Resilience indicators subsection in the Methods to provide more details on the calculation of resilience indicators (see the added paragraph below). As the calculation of resilience indicators results in a sequence of calculation of well-known statistical concepts, such as variance, autocorrelation or PCA, we do not provide the formulas. We used the package generic_ews for Matlab (https://git.wur.nl/sparcs/generic_ews-for-matlab), a published open-source package for the calculation of resilience indicators which provide step by step functions to reproduce our results (1). It is based on a similar package in R that is widely used (2).

Lines 262-272: "*We calculated all resilience indicators using the rolling window method for time series as described in Dakos et al. (37). For this method, we need to detrend the data to avoid spurious increase of the indicators due to their trend. We used a Gaussian kernel with the optimal bandwidth according to (Bowman & Azzalini 1997), for this (formula provided in Supporting Information). A rolling window of 50% of the size of the detrended time series is used to calculate the indicators and assess their trend over time: in each window, the value of each indicator is calculated (for univariate indicators, using the built-in functions to calculate variance and autocorrelation in Matlab) (37). We then evaluate the strength of the trend in the indicators over time by calculating the Kendall tau correlation between the value of each indicator in each window and time. We use the open-source tool generic_ews for Matlab (https://git.wur.nl/sparcs/generic_ews-for-matlab) (39).*"

4. Another limitation which I believe needs to be acknowledged is that in your simulations R_0 reaches the threshold value 1 in about 8 years, whereas in reality this occurs within a few weeks between spring and summer.

Thank you for your comment. Indeed, this scenario is a highly simplified representation of reality, but constitute a first step in the study of multivariate indicators of resilience. The between-year increase in R_0 is chosen to reflect the speed of climate-change drive increase in the average R_0 as predicted by (3). Seasonal fluctuations are omitted as a simplification, but previous research found that they have limited impact on the prediction performance of univariate resilience indicators, even when the amplitude of the seasonal fluctuations is as large as the amplitude of the between-year increase (4). Further research will require to test if these findings hold for multivariate indicators, and how the monitoring scenarios can be implemented to ensure that signals can still be observed.

We have clarified this choice in the method section, lines 208-215: "*The biting rate varies over time to alter the R_0 and, thus, the resilience of the system. The biting rate is known to be influenced by temperature, which mimics a simplified representation of the emergence of WNV*

due to increasingly suitable climatic conditions over the years (5). Other parameters affected by the temperature, such as the extrinsic incubation period or mosquito birth and death rates, are kept fixed to avoid interactions between the parameters obscuring the dynamics of emergence, and thus our understanding of the indicators. For the same reasons, seasonal fluctuations were omitted and the emergence of WNV was modelled as being driven solely by the between-year increase of the average R_0 associated with climate change."

We have amended the discussion to show this limitation, lines 469-473: "*Further, seasonal fluctuations were omitted, and the emergence of WNV was driven only by a gradual increase of R_0 over several years. Previous research with univariate indicators found a limited impact of seasonal fluctuations on the prediction performance of resilience indicators, even for fluctuations of high amplitude relative to the between-year increase (4). Similarly, overdispersion was omitted in this model but previous research found that it has a limited impact on the performance of resilience indicators (6). Although indicators of resilience loss hold potential to improve the current practice of early warning for vector-borne diseases, these findings should be confirmed in datasets generated with complex models, but also real data specific to a certain pathogen.*".

Minor points

1. Lines 49-51. Check this sentence.

Thank you for pointing it out. The sentence has been rephrased as *"The transmission of VBD involves several species interacting with one another, which can be monitored through different data sources."*

2. Please describe WNV upon its first mention (line 79, you can move lines 114-116).

Thank you for your comment. We have now rephrased as: *"For instance, West Nile virus, an enzoonotic disease transmitted between birds and mosquitoes but also affecting horses and humans as dead-end hosts, can be monitored in numerous ways. Transmission can be monitored in mosquito pools by estimating the proportion of infected mosquitoes or the proportion of infected pools (7), but also by monitoring infections in humans, livestock, or wildlife through public health and veterinary surveillance systems (8)."*

3. Lines 120-122. In the case of WNV, birds are also monitored by trapping and shooting them, see for instance [1].

Thank you for your comment. We have amended the manuscript accordingly: *"Monitoring live birds is also possible but requires a greater effort as they must be caught and sampled by bird ringers or citizen scientists, e.g. (22–24), and is thus more costly than sampling dead birds. Alternatively, hunting or trapping also allows for live bird sampling (26)."*

4. Since you assume that all mosquitoes are non-diapausing ($\delta M=1$) you might consider removing this parameter to simplify the model.

We have now removed this parameter from the model equations to simplify the model.

5. Figure 1A. Since visible birds can either die or recover from the infection, I think there should be two arrows connecting BVI to BVR or BVD.

Thank you for pointing out that mistake. We have updated Figure 1 accordingly.

6. Please provide the unit (daily?) of the rates shown in Table S1.

We have amended Table S1 accordingly.

7. Line 193. I assume you mean absence of infected mosquitoes and birds, please remark this (you might still have WNV circulating in mosquitoes but have no infectious birds for some time).

Thank you for pointing out that unclarity. We have rephrased as: *"(…) the recovery time to the disease-free equilibrium (i.e. the absence of infectious birds, resp mosquitoes) is calculated based on the exponential decay of infectious individuals after the perturbation."*.

8. Lines 290-292. I understand that if the indicator signals a positive trend but $R_0<1$ then this is a false positive, am I right?

It is correct that if the indicator displays a positive trend but R_0 is stable <1, then this is a false positive. We have rephrased to "*Emergence time series with increasing R_0 leading to an epidemic are used to calculate the true positive rate and false negative rate, while time series with a fixed R_0 with no upcoming epidemic are used to calculate the true negative and false positive.*" to improve clarity.

9. Figure 2. Are these single stochastic simulations?

In figure 2A, yes these are single stochastic simulations displayed as an example, we have added it in the figure caption to improve clarity. In Figure 2B and 2C, the recovery time is calculated over 1000 repetitions of stochastic simulations, we have added the confidence interval as requested by reviewer #3 and clarified the method section.

Lines 196-198: "*We perform these experiments for both birds and mosquitoes, over 1000 replicates per R_0 value to estimate the average and the confidence intervals of the recovery time.*"

Lines 316-323: "*Fig 2. (A) Number of infected birds A over time, for two stochastic simulations as examples of the perturbation-recovery experiments in a case of high (red) and low (blue) resilience. The points represent the observations of infected birds over time, and the dotted lines indicate the fitted lines used to calculate the return rate to the disease-free state (see Methods). (B) Recovery time (in days) to the disease-free state after perturbing the system by introducing infected birds for different values of R0. (C) Recovery time (in days) to the disease-free state after perturbing the system by introducing infected mosquitoes for different values of R0. For B and C, the recovery time is defined as the reciprocal of the return rate, assuming exponential decay.*"

Is recovery time expressed in days?

Yes. We have added (in days) to the figure caption.

10. As WNV transmission is affected by several environmental drivers, there are many abiotic indicators (e.g. spring temperature) which might provide some kind of early warning indicator even before detecting the virus, which conversely is needed to use the indicators proposed in the manuscript. Please add some text to discuss this, with some references (e.g. [2–4]).

Thank you for your comment. We have amended the discussion to include a paragraph on alternative early-warning approaches, lines 510-516:
"*Finally, this study focused on the use of resilience indicators as an anticipation method, which is based solely on epidemiological time series and require prior detection of the virus. Although resilience indicators hold the advantage of being generic and model-free, and thus do not require model-fitting, other methodologies have been developed relying on environmental drivers such as temperature and precipitation as an early-warning indicator for West Nile virus outbreaks as well as for other mosquito-borne disease outbreaks (52-54). A combination of such methodologies with resilience indicators could be envisioned to leverage the advantages of both methodologies.*".

Bibliography
1. De Nardi A, Marini G, Dorigatti I, Rosà R, Tamba M, Gelmini L, et al. Quantifying West Nile virus circulation in the avian host population in Northern Italy. Infect Dis Model. 2025;10: 375–386. doi:10.1016/j.idm.2024.12.009
2. Marini G, Manica M, Delucchi L, Pugliese A, Rosà R. Spring temperature shapes West Nile virus transmission in Europe. Acta Trop. 2021;215: 105796. doi:10.1016/j.actatropica.2020.105796
3. Farooq Z, Rocklöv J, Wallin J, Abiri N, Sewe MO, Sjödin H, et al. Artificial intelligence to predict West Nile virus outbreaks with eco-climatic drivers. Lancet Reg Health - Eur. 2022;17: 100370. doi:10.1016/j.lanepe.2022.100370
4. Moirano G, Fletcher C, Semenza JC, Lowe R. Short-term effect of temperature and precipitation on the incidence of West Nile Neuroinvasive Disease in Europe: a multi-country case-crossover analysis. Lancet Reg Health - Eur. 2025;48: 101149. doi:10.1016/j.lanepe.2024.101149

Reviewer #2:
Review report – Multivariate resilience indicators to anticipate vector-borne disease outbreaks: a West Nile virus case-study.

1. **Article summary**

The article examines and evaluates various resilience indicators (RIs), which are statistical indicators used to detect early warning signs of critical transitions in complex systems, in the context of epidemiological surveillance. The study focuses on the application of RIs to vector-borne diseases, with the goal of improving early warning systems for disease outbreaks. To achieve this, the authors conduct a simulation study on West Nile Virus, a mosquito-borne virus that is expected to become more prevalent in Europe due to favorable climate change conditions. The authors aim to assess the performance of RIs, which are based on the idea that systems approaching a tipping point will exhibit slowing recovery times i.e. it will take longer to return to its normal state after a perturbation. In the

context of disease epidemiology, this means that RIs can potentially detect early warning signs of an outbreak emergence, which occurs when the basic reproduction number (R0) approach 1.

The authors demonstrate that in this case-study variance-based RIs outperform autocorrelation-based ones, and that multivariate RIs are more effective, especially when dealing with low-resolution data. Contrary to previous findings, the authors show that in this case study, there is no evidence to suggest that one species is more informative than another for early warning purposes, despite their respective contributions to the transmission cycle.

2. General assessment:

As someone who was not familiar with RIs and critical slowing down theory prior to reading this article, I found the authors' explanation and application of these concepts clear and well-motivated. After conducting some brief review of the literature and analyzing the provided information, in my opinion the article is a well-structured and well-written research paper that provides an original contribution to the field regarding the use and evaluation of resilience indicators as a model-free tool for early warning systems in the context of West Nile Virus and, more broadly, vector-borne diseases. Through their analysis, the authors effectively demonstrate novel results: that variance-based RIs outperform autocorrelation-based ones, multivariate RIs outperform univariate indicators and are more robust to low-resolution data. Moreover, the authors showed that no species in this system is more informative for early warnings whether it is more involved in the transmission cycle. These findings have significant implications for real-life applications, such as the choice of strategy for data collection in the setup of an early warning system for West Nile Virus outbreak surveillance. For instance, the use of multivariate RIs could enable more effective surveillance, ultimately leading to improved disease outbreak prevention and control.

However, some aspects of the article require clarification or improvement, particularly regarding the precise methodology used to evaluate the performance of the RIs i.e. the classification of positive outbreaks and negative outbreaks in the computation of the AUC and at which temporal horizon. Providing more detailed information on this aspect would strengthen the article and enhance its overall validity.

3. Strengths:

- The paper is clear and didactically written, facilitating understanding.
- Figures are neat, easy to interpret and visually appealing.
- The use of a simulation study is particularly relevant in this context, allowing precise control over the elements of the epidemiological system. This approach enables an insightful exploration on how the different RIs perform across the different scenarios and data resolutions.
- The methodology used, rooted in critical slowing down theory seems robust and have been already validated.
- Scenario simulations with varying data streams and resolutions effectively illustrate that multivariate indicators outperform univariate indicators, especially in conditions of low data resolution. This result has significant implications for practical surveillance and supports initiating discussions on real-world implementations of surveillance strategies for early warning system.

Thank you for your careful review. We are happy that you found our article useful and easily understandable. Consistent with the comments of the other reviewers as well, we have improved the method section to ensure that the readers can follow the step-by-step methodology and reproduce our analyses. We respond to your comments one by one in the text below.

4. **Detailed comments:**

Major comments:

- It appears that a crucial methodological detail is missing regarding the evaluation of RIs' performance. While the authors state they used the AUC of the ROC curve based on the classification of true positives and true negatives at different Kendall tau cut-off values derived from simulation of emergence time series (upcoming epidemic vs. no upcoming epidemic), the article lacks specifics on how this classification was precisely conducted. Clarification is needed on how the data was generated, what decision rules were applied, and what temporal horizon was used for outbreak predictions. Without this information, it is difficult to assess the validity and practical applicability of the RIs, particularly in the context of an early warning system.

Thank you for your comment.
The data are generated using the model explained in the Model subsection of the methods, we have improved that subsection based on the comments of the other reviewers (see corresponding comments in the rebuttal letter). The full generated time series (from R_0=0.7 to R_0=1 for emergence time series) are used for the classification. We have clarified that temporal horizon in line 227: "*In these time series, R_0 gradually increased from 0.7 to 1 over 428 weeks with a weekly resolution (Fig 1B). As a control, we simulate "stable time series" under the assumption of a fixed R_0 over time, leading to no major outbreaks (Fig 1C). We use R_0=0.8 for that scenario. We simulate 100 stochastic repetitions for each R_0 condition, and the full simulated time series were used in the analyses.*"

Regarding the assessment of the indicators' performance using ROC curves, we have amended the Methods to provide more details, lines 296-304: "*We quantify the indicators' performance as early-warning signals using ROC (receiver operating characteristic) curves. Emergence time series with increasing R_0 leading to an epidemic are used to calculate the true positive rate and false negative rate, while time series with a fixed R_0 with no upcoming epidemic are used to calculate the true negative and false positive. The ROC curve is calculated based on the list of Kendall tau values labeled with the outcome (upcoming epidemic or not), using the perfcurve function of the Statistics and Machine Learning toolbox in Matlab. The Area Under the ROC Curve (AUC) was used to estimate the prediction performance of the different indicators under different data scenarios over 200 repetitions, 100 emergence time series and 100 fixed time series.*"
Additionally, and based on the other reviewers' comments, we have included more details in the method section about the calculation of the resilience indicators in lines 279-291.

Minor comments:

- The introduction could gain clarity from minor structuring. For example, the authors introduced West Nile Virus on Line 79, but reintroduce it Line 114. Additionally, on

Line 98 the authors stated that multivariate indicators have not been compared to univariate indicators, and they restate the same claim Line 111.

Thank you for your comment, consistent with the comment of reviewer #1. We have now revised the introduction by defining West Nile virus upon its first mention, line 80: "*For instance, West Nile virus, an enzoonotic disease transmitted between birds and mosquitoes but also affecting horses and humans as dead-end hosts, can be monitored in numerous ways.*".
Additionally, we have removed the repetition mentioned in line 111.

- The « data poor » scenarios assume regular sampling intervals. It would be beneficial, at least briefly, to discuss or assess how irregular sampling (data points missing-at-random) or varying sampling frequencies across hosts (in multivariate indicators settings) might impact RIs performances.

Thank you for your comment. For autocorrelation-based indicators, regular sampling interval are necessary. When this is not the case, a preprocessing methodology consists of using interpolation to obtain regular sampling intervals in the dataset. We have mentioned this in the discussion, lines 450-454:
"*Additionally, the performance of autocorrelation-based indicators decreased when the resolution of the time series was reduced, in agreement with previous studies (9,10) (Fig S4 to S6). Further, we kept regular sampling intervals in our analyses, as this is necessary for the calculation of autocorrelation. Irregular sampling interval would require interpolation which would probably further reduce the performance of autocorrelation based-indicators.*".

- Line 337: the authors stated that autocorrelation based RIs perform worse than variance one due to the scarcity of cases resulting in highly variable autocorrelation due to long stretches of 0. Looking at figure 1, I see how it could be the case for horses for example but is this equally true for bird and mosquito incidence? This warrant clarification.

Figure 1 shows aggregated data into monthly reports, leading to less zeros. However, in the original dataset used for the analysis, long stretches of zeros are present for low R_0 values, both at the start of emergence time series and for the whole fixed time series, not only for horses and humans, but also for mosquitoes and birds.

We have clarified in the text, lines 185-186: "*In (B) and (C), the time series represent a single simulation and were aggregated per month to improve the readability of the figure, but the daily time series are used in the analyses.*"

And lines 351-352: "*Due to the scarcity of cases far from the epidemic threshold, the time series of all hosts and vectors contained long stretches of consecutive zeros, resulting in a highly variable autocorrelation, which altered the performance of autocorrelation and autocorrelation-based indicators (Fig S1 to S3).*"

- Line 340: After the results of the performance of the RIs, authors choose to continue the analyses with the hidden bird reservoir incidence or seroprevalence and in the case of the multivariate RIs with the hidden reservoir scenario. Given that, by

definition, this reservoir is hidden, evaluating observational probability or down sampling on this data source seems somewhat contradictory. Especially when you consider that for the three best performing multivariate RIs, the wildlife scenario is on par with the hidden reservoir scenario performance. Clarification of intent or reconsidering the data source maybe beneficial.

Thank you for your comment. By “hidden”, we mean that it is harder to observe since few birds of that species die from the infection, as was observed for instance for Usutu virus (11). However, using traps, infections and seroprevalence in the “hidden” bird species can be reported, and are likely to be subject to underreporting as this species does not experience as many symptoms as the visible one. As the hidden reservoir scenario yields the best performance and since we have to choose one scenario in the main text, we proceed with the best scenario in the rest of the analyses. However, we believe that the results of the analyses are of interest for all the scenarios, hence they are all provided in the SI. Additionally, the results are consistent across all the scenarios, as stated in the main text.

We have clarified what we mean by “hidden reservoir” in the methods lines 153-156: “*Bird species H is a hidden reservoir: it is preferred by mosquitoes but it does not experience symptoms and does not die from infection, hence complicating the detection of transmission in this species through dead bird surveillance. However, both bird species are equally hard to monitor through live bird surveillance.*”

And in the discussion lines 445-448: *“Answering this question is especially relevant for the surveillance of various infectious diseases, especially when silent transmission is ongoing in hidden reservoirs that are harder to observe as they do not experience symptoms and deaths, and have to be monitored through live bird sampling (31,45).”*

- Line 416: The authors stated that « The prediction performance of the different multivariate monitoring scenarios, as well as univariate time series, remained stable regardless of how much a species is involved in the transmission cycle. Noticeably, increasing the mosquito feeding preference towards a given species or the relative abundances of the different species did not increase the potential for that species to signal upcoming outbreaks » which they say contradict earlier findings. However, the explanation provided -high spillover rates to dead-end species- appears inadequate. Looking at Figure 5A and S13, the performance of univariate variance-based RI is comparable across different species regardless of incidence (e.g. horse incidence). Moreover, this hypothesis can be tested with further analysis, such as sensitivity analysis of human-or-horses-based RIs performance with respect to these parameters. Therefore, further investigation is needed to fully understand this result.

Thank you for your comment. We agree that this result was surprising, and despite running some additional analyses (that we did not include in the manuscript) to investigate if some properties of the time series drive the prediction performance of resilience indicators, we could not find a satisfactory explanation (Figure I).

These analyses were run similarly to the analyses presented in the manuscript for Figure 5B: calculating the AUC of the univariate indicators for the different univariate time series generated when varying the biting preference of the mosquitoes towards the hidden bird reservoir. However, here we investigate the effect of intrinsic properties of the time series: the number of zeros in the time series (Figure I A and B), the amplitude of the time series, calculated as *maximum y – minimum y* (Figure I C and D), the maximum value in the time series (Figure I E and F) and the sum of all the values in the time series (Figure I G and H).

For instance, a species with a relatively low involvement in the transmission cycle would exhibit a high number of zeros due to the rare observation of cases (Figure I A and B), whereas a species with a high involvement would exhibit a high maximum in the time series (Figure I E and F) and a larger amplitude (Figure I C and D). However, none of these processes seem to influence the prediction performance quantified using the AUC. Further analyses are required to understand what really drives how informative a data source is.

However, we believe that really low spillover rates combined with low observation probability would decrease the prediction performance as no cases at all would be observed, making the calculation of resilience indicators impossible. To improve the clarity of the discussion, we have rephrased this statement as "*The model assumed relatively high spillover rates to dead-end species leading to cases in dead-end species that are observed even with low reporting probabilities. However, this is not always the case in practice and could hamper the prediction performance of resilience indicators when no cases are detected (41).*".

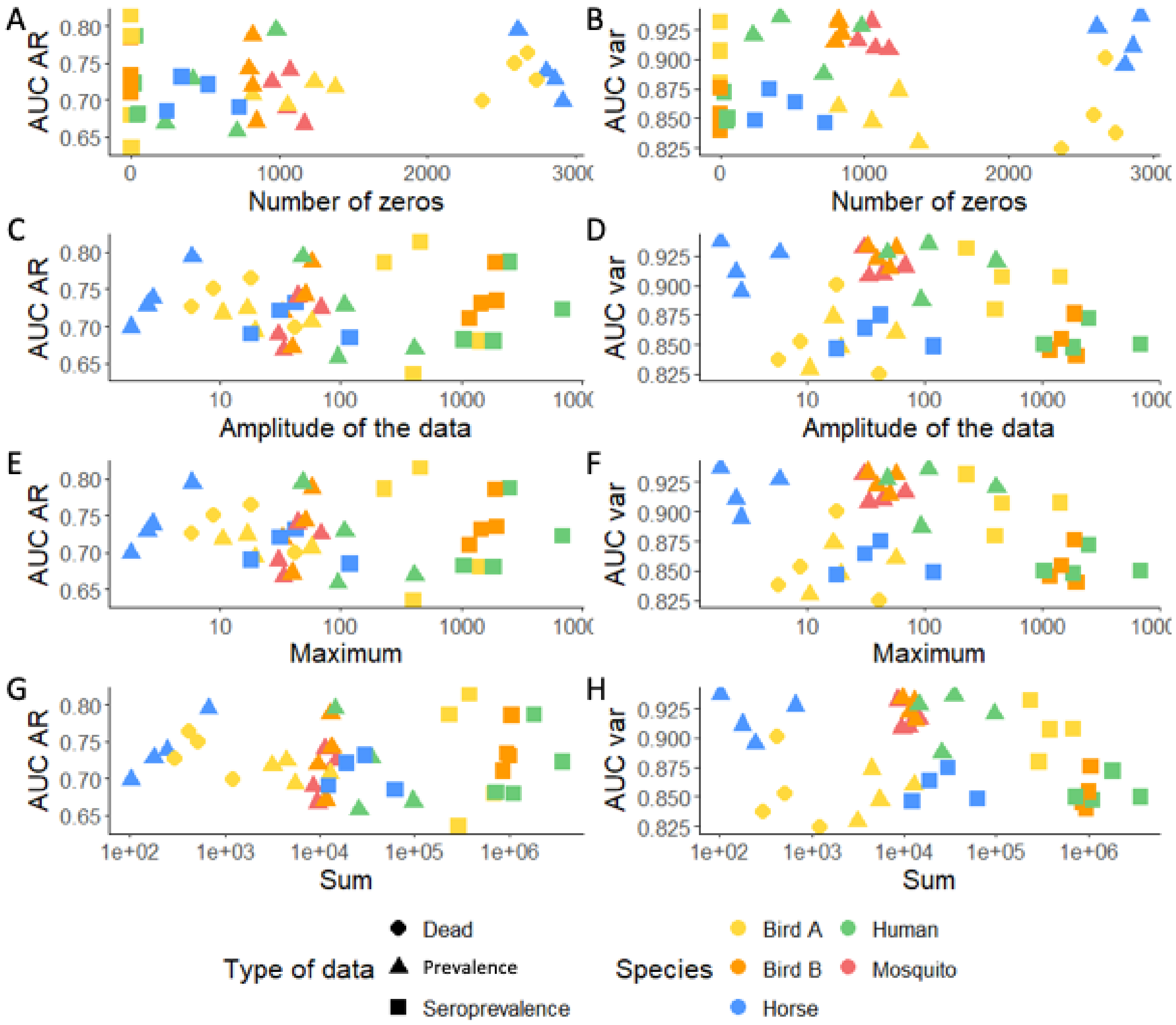

<u>Figure I</u>: Performance of univariate resilience indicators for different properties of the time series. Autocorrelation (A, C, E, F) and variance (B, D, F, H) are evaluated using the AUC. Effect of the number of zeros (A, B), amplitude of the data defined as *max-min* (C, D), maximum in the time series (E, F) and total sum of the time series (G, H) on the performance of the indicators.

5. Suggestions:

- Figure 1: Panel readability could improve by decreasing panel A's size and enlarging panel B and C because plots are difficult to read in these. Additionally, including plots of variance and autocorrelation of incidence or seroprevalence time series here might clarify RIs' behaviors visually. Also, for the two bottom plots, the legends are not consistent between left and right plots.
- Figure 2: Clearly indicate the recovery time unit.
- Figure 3: Consider adding a y-axis for each panel or using a darker grid for improved readability.
- Line 343: Likely « human seroprevalence » rather than prevalence.
- Line 356 and 358: Panels should be ordered in the same sequence as they appear in the text i.e. 4A and 4B; 4C and 4D.
- Line 361: Rephrase the sentence to improve clarity, for example: « Multivariate indicators demonstrated greater robustness than univariate indicators,

maintaining higher performance despite reductions in data resolution and observation probability »

- Line 442: Elaborate briefly on why system reactivity could influence variance performance.
- Line 444: This sentence in unclear especially considering the previous statement that several sources of data require increased collection efforts. It may need rephrasing to clearly communicate intended meaning.

Thank you for all these suggestions. We have incorporated them in the manuscript.

6. **Questions for the authors:**
    - Figure 1: Is it expected behavior for bird hosts to have higher dead incidence than seroprevalence at certain points in the simulation (e.g. panel C near the end)?

For the visible bird species, 30% of infected birds die. Additionally, in the compartment of recovered birds decreases because of natural death of the birds, whereas the compartment of dead birds only increases over time.

- Figure 3, 4, 5: Could you confirm whether each AUC points represent the classification performance across 200 simulations (100 with increasing R_0 and 100 with stable R0) for each cut-off of Kendall tau?

The AUC are indeed calculated across 200 simulations, split equally between data with upcoming epidemic and data without an upcoming epidemic.

- Figure 5B: Could you explain what variable changes between points the same data type and species (for example the several blue triangles)?

The change in the typical infection coefficient is simulated as a change of biting preference toward bird V. However, for horses that are already an unpreferred species and also not densely present, the change of biting preference towards the birds does not lead to a great change in their typical infection coefficient, hence the several almost overlapping blue triangles.

- Given that you maintain the R_0 within 0.7 – 1.0 by adjusting biting rates when varying mosquito feeding preference towards hidden birds, have you verified that bite rate remain biologically plausible under extreme preference scenarios?

Thank you for pointing that out. The biting rate remains in a plausible interval (from 0.2 to 0.92), consistent with WNV parameter values retrieved in (12).

7. **Recommendations**:
    Based on these comments, a minor revision is recommended to address methodological clarification (especially the RIs performance assessment methodology) and minor comments.

Reviewer #3:

This manuscript addresses a central problem of infectious disease surveillance—low and biased reporting—which may be particularly severe in multi-host vector-borne systems such as West Nile virus (WNV). The authors approach this issue using ideas from complex systems and time-series analysis related to signals of resilience. Specifically, the manuscript tests the use of multivariate resilience indicators using a model-generated synthetic dataset for WNV.

A key result, to me, is that multivariate indicators outperformed univariate indicators when sampling resolution and observation probability were reduced.

Thank you for your comments. We respond to your suggestions one-by-one below.

Major Comments

1. I suggest more technical precision and rigor in defining indicators of resilience. For example, around line 71, autocorrelation and variance are introduced as resilience indicators. What is the statistical test for slowing down when calculating variance and autocorrelation statistics in a time series over a moving window? Similarly, the sentence beginning on line 90 about multivariate indicators of resilience could use a mathematical definition or a "For instance…"

Thank you for your comment, we have clarified both sentences.

About resilience indicators, lines 70-73: "*This slow behaviour can be observed in trends over time of so-called resilience indicators, namely autocorrelation and variance (13). Such trends are usually evaluated in the prevalence time series using a rolling window, and their significance can be assessed by calculating Ebisuzaki p-values (14).*"

About multivariate resilience indicators, line 95: "*When data from multiple sources are available, the information they contain can be combined to calculate multivariate indicators of resilience, for instance mean autocorrelation and variance (11).*"
Additionally, in line with other reviewers' comments, we have clarified the Method section to provide a rigorous step-by-step description of the calculation of resilience indicators.

2. I'd like to see the differential equations for the model at the top of the Methods. "Adapted from (28)" isn't helpful—even if the reader is familiar with reference 28—because we don't know what "adapted" means. Just state the model.

Thank you for your comment. Due to the number of equations and to keep a reasonable length of the manuscript, we have provided the equations in the Supplementary Information. However, extra attention was paid in providing a clear description of the model in the Methods, lines 146-154.

"*We use a well-known compartmental Ross Macdonald model of WNV for which the dynamics are well-studied (31). Our model considers the vector population and four host populations: two birds of generic species acting as amplifying hosts, and humans and horses acting as dead-end hosts (Fig 1A, equations provided in Supporting Information). The two bird species differ in how they are affected by the disease. Bird species V is a visible, i.e. detectable, reservoir for the disease: 30% of the infected individuals die from infection (as can be the case for corvids, for instance*

(32,33)). Bird species H is a hidden reservoir: it is preferred by mosquitoes but it does not die from infection, hence complicating the detection of transmission in this species through dead bird surveillance. The model is used to generate time series."

3. For variance as an indicator, how does the approach deal with mean-variance scaling? How does this approach perform at different population sizes? What happens to the differential performance of multivariate versus univariate resilience indicators as population sizes become very small or very large? (By manipulating population size, I mean manipulating the overall magnitude of the time series.)

Indeed, many things can have an effect on variance, for instance the amount of noise, whether it is autocorrelated and the mean value (if the noise is multiplicative). Therefore we only compare the indicators within the same time series, by calculating the correlation with time, where we assume that these effects stay constant. Moreover we conducted a sensitivity analysis varying the relative abundances of bird V and bird H, influencing the magnitude of the time series (Supporting Information, Figures S21 to S25). In our case, the results were consistent across the tested population abundances.
Additionally, as specified in the response to the last minor comment of reviewer 2, exploratory analyses found no effect of the amplitude of the time series on the prediction performance of univariate indicators (Figure I).

4. The monitoring scenarios assume a Poisson distribution. But infectious disease incidence data is often overdispersed and conforms better to a negative binomial distribution. I would be curious how the results change when using a negative binomial distribution, especially across different levels of overdispersion.

Thank you for your comment. We agree that incidence data are often overdispersed and that a negative binomial distribution could improve the model. However, we decided to keep a Poisson distribution to limit the number of parameters in the model and limit the factors potentially affecting the performance of our indicators. The effect of overdispersion on the performance of (univariate) indicators of resilience has been explored in (15) and is now mentioned in the discussion, line 476 "*Similarly, overdispersion was omitted in this model but previous research found that it has a limited impact on the performance of resilience indicators (45).*".

5. The perturbation-recovery experiments measure the rate of return to the DFE in an exponential model. But the introduction focuses on using variance and autocorrelation to measure resilience. Sorry if I missed it, but I suggest the authors add something to the introduction to discuss this return-time measure. The authors may be interested in this paper, which describes a related approach and could serve as a useful complement to the next-generation method for calculating R_0:
Neubert, M. G., & Caswell, H. (1997). Alternatives to resilience for measuring the responses of ecological systems to perturbations. Ecology, 78, 653–665.

Thank you for your suggestion. As stated lines 68-70, resilience indicators (such as autocorrelation and variance) *"rely on the theory of critical slowing down, stating that a system*

approaching a critical transition, such as the start of an epidemic, loses its resilience and recovers more slowly from external perturbations (3-6)".
Autocorrelation and variance are proxies for measuring a loss of resilience, while the recovery time measured in the perturbation-recovery experiments are direct measures of the resilience. Thus, the perturbation-recovery experiments are run to control for the presence of critical slowing down in the system before going further, as now stated line 187: "*To study the presence of critical slowing down in this system, we perform perturbation-recovery experiments using the WNV model described above for different values of R_0 (i.e. the basic reproduction number, defined as the number of secondary cases expected from an infectious individual in a fully susceptible population).*".

6. Figure 2: I was expecting averages and credible intervals over replicate simulations. I suggest the authors include those and/or clarify what the figure shows and why, particularly with respect to statistical inference. Based on what's currently shown, it seems likely the replicates behave similarly, but this should be stated.

Thank you for your comment. We have now added the confidence intervals for Figure 2B and 2C. We have also clarified the method section and the figure caption.

Lines 197-199: "*We perform these experiments for both birds and mosquitoes, over 1000 replicates to estimate the average and the confidence intervals of the recovery time.*"

Figure caption "*Fig 2. (A) Number of infected birds A over time, for two stochastic simulations as examples of the perturbation-recovery experiments in a case of high (red) and low (blue) resilience. The points represent the observations of infected birds over time, and the dotted lines indicate the fitted lines used to calculate the return rate to the disease-free state (see Methods). (B) Average recovery time (solid line) and 95% confidence interval (dotted line) (in days) to the disease-free state after perturbing the system by introducing infected birds for different values of R0. (C) Average recovery time (solid line) and 95% confidence interval (dotted line) (in days) to the disease-free state after perturbing the system by introducing infected mosquitoes for different values of R0. For B and C, the recovery time is defined as the reciprocal of the return rate, assuming exponential decay, and is calculated over 1000 replicates.*".

7. Figures 4 and 5: I'm not sure how to interpret changes in AUC as a function of variables such as sampling resolution and feeding preference. Why do AUC values fluctuate—for example, increasing slightly rather than decreasing—when sampling resolution goes from weekly to biweekly? When should such fluctuations be interpreted as meaningful trends, and when are they considered negligible? This part of the analysis may require a clearer statistical framework and potentially more replicate simulations.

Thank you for your comment. Due to time constraints and limited computational power, we decided to stick with 200 replicates (100 emergence time series and 100 stable time series) throughout the analysis, as it yielded robust results across the different analyses. As stated in the text, further research is necessary to find what drives how informative a data source is, and what could explain the fluctuations observed in Figure 4 and 5. Additionally, as stated in a similar comment of reviewer #2, additional explorative analyses (not included in the

manuscript) could not provide answers to that question. Although we think this is an important question, our analyses only provide initial answers (what does not drive how informative a source is). We think however that this is an important result as it contradicts previous findings, and leaves room for further research dedicated to that question.

Minor Comments

Line 69: “recovers slower” → “recovers more slowly”

Done.

There's a discontinuity between paragraphs 1 and 2 of the Introduction. The first is about resilience indicators and critical slowing down; the second jumps to multi-disease surveillance. Consider adding a transitional sentence to bridge these topics.

As a bridge, we have added the sentence (lines 75-77):
“*However, the large data requirements limit their use in practice, it is thus necessary to design appropriate monitoring strategies consistent with the disease system of interest.*

Surveillance of multi-host diseases is challenging to achieve, as many, sometimes unknown, species can participate in the transmission, which leaves choices on which sources to sample from (8). "

The paragraph starting on line 102 is important in making the case for multivariate resilience indicators for monitoring complex systems but needs some tightening. The sentence on line 104 could be removed or revised; complex systems by definition have multiple interacting components, and there is almost always a choice of where to direct monitoring. Similarly, the next sentence—"complex systems must have these types of interactions"—could be made more precise or omitted.

Thank you for your comment. To improve the precision but keep the flow of the paragraph, we have rephrased both sentences as: “*Designing monitoring strategies for complex systems is complicated by the numerous entities interacting, sometimes with poorly understood interactions (20).*"

The Methods section switches to past tense after the first paragraph. I suggest using present tense throughout for consistency.

Thank you. We have made the correction.

The term “generic bird” doesn’t have a clear biological meaning. Consider omitting “generic” or replacing with something like “representative bird species” or simply “bird.”

We clarified as “*two representative bird species acting as amplifying hosts*”

Line 177: The discrete events simulated by a Gillespie algorithm do not necessarily map to a branching process. Regarding why the Gillespie algorithm is used, I suggest wording like: “to

model stochasticity arising from random variation in the timing of infection and removal events," and omitting the reference to branching processes.

We have revised the manuscript as suggested: *"Intrinsic stochasticity arising from random variation in the timing of events is added to the model using the Gillespie algorithm (37).".*

Line 261: For reproducibility, I suggest stating the optimal bandwidth used for detrending the emergence time series, rather than citing a prior paper without specifics.

Thank you for your comment. We have clarified that paragraph, lines 263-267: "*We calculated all resilience indicators using the rolling window method for time series as described in Dakos et al. (11). For this method, we need to detrend the data to avoid spurious increase of the indicators due to their trend. We used a Gaussian kernel with the optimal bandwidth according to (41) for this (formula provided in Supporting Information).*"

We have added the formula to calculate the optimal bandwidth in the Supplementary Information: "*We calculated all resilience indicators using the rolling window method for time series as described in (7). For this method, we need to detrend the data to avoid spurious increase of the indicators due to their trend. We used a Gaussian kernel with the optimal bandwidth according to (41), as provided in the package generic_ews in Matlab (https://git.wur.nl/sparcs/generic_ews-for-matlab).*

The optimal bandwidth h is calculated using

$$hx = \frac{median(\,|x - medx|\,)}{0.6745}\,\frac{\frac{4}{3}}{n}^{0.2}$$

$$hy = \frac{median(\,|y - medy|\,)}{0.6745}\,\frac{\frac{4}{3}}{n}^{0.2}$$

$$h = \sqrt{hy \cdot hx}$$

with x the time and y the time series, and medx and medy the median of x and y respectively. "

Line 267: I suggest listing the indicators used at the top of the paragraph before discussing them in more detail. Many readers may not be familiar with the set used by Weinans et al. (2021).

We have added a list of indicators, line 274: "*We use a subset of the multivariate indicators described in (15): mean autocorrelation and variance, maximum autocorrelation, covariance,*

cross-correlation and variance, PCA and MAF autocorrelation and variance, and PCA eigenvalue and MAF eigenvalue.".

The citation style shifts from numeric to author-date format in the Discussion. This should be made consistent.

Thank you, we have revised the manuscript accordingly.

Line 325: What does "no meaningful difference" mean statistically? Please clarify the basis for this claim.

As we have not tested the significance statistically, we have clarified by specifying the value of the difference "*Although the Hidden reservoir scenario yielded the best prediction performance across all indicators, there was a small difference in the average AUCs of the different multivariate scenarios, ranging between 0.80 and 0.83.*".

Line 408: I suggest avoiding priority claims like "Our study was the first…" These are difficult to verify and don't strengthen the results or their interpretation. Instead, focus on the specific contributions and implications for epidemic modeling and surveillance.

We have rephrased as "*As data requirements limit the use of resilience indicators in practice, these findings could have important implications for the surveillance of vector-borne diseases.*".

References

1. Scheffer M, Van Nes EH, Bird D, Bocinsky RK, Kohler TA. Loss of resilience preceded transformations of pre-Hispanic Pueblo societies. 2021;118(18).
2. Dakos V. Earlywarnings in R. 2015.
3. Erazo D, Grant L, Ghisbain G, Marini G, Colón-González FJ, Wint W, et al. Contribution of climate change to the spatial expansion of West Nile virus in Europe. Nat Commun [Internet]. 2024 Feb 8;15(1):1196. Available from: http://www.ncbi.nlm.nih.gov/pubmed/38331945
4. Miller PB, O'Dea EB, Rohani P, Drake JM. Forecasting infectious disease emergence subject to seasonal forcing. Theor Biol Med Model [Internet]. 2017;14(1). Available from: internal-pdf://236.4.215.153/Miller-2017-Forecasting infectious disease eme.pdf
5. Paz S, Semenza JC. Environmental drivers of West Nile fever epidemiology in Europe and Western Asia--a review. Vol. 10, International Journal of Environmental Research and Public Health. Multidisciplinary Digital Publishing Institute (MDPI); 2013. p. 3543–62.
6. Brett TS, O'Dea EB, Marty É, Miller PB, Park AW, Drake JM, et al. Anticipating epidemic transitions with imperfect data. PLoS Comput Biol. 2018;14(6).
7. Engler O, Savini G, Papa A, Figuerola J, Groschup MH, Kampen H, et al. European Surveillance for West Nile Virus in Mosquito Populations. German Mosquito Control Association (KABS) OPEN ACCESS Int J Environ Res Public Health [Internet]. 2013 [cited 2023 Jun 27];10:10. Available from: www.mdpi.com/journal/ijerph

8. Habarugira G, Suen WW, Hobson-Peters J, Hall RA, Bielefeldt-Ohmann H. West nile virus: An update on pathobiology, epidemiology, diagnostics, control and "One health" implications. Vol. 9, Pathogens. MDPI AG; 2020. p. 1–51.

9. Weinans E, Quax R, van Nes EH, Leemput IA van de. Evaluating the performance of multivariate indicators of resilience loss. Sci Rep. 2021 Dec 1;11(1).

10. Dakos V, Van Nes EH, D'Odorico P, Scheffer M. Robustness of variance and autocorrelation as indicators of critical slowing down. Ecology [Internet]. 2012 Feb 1 [cited 2022 Jul 6];93(2):264–71. Available from: https://onlinelibrary.wiley.com/doi/full/10.1890/11-0889.1

11. de Wit MM, Beaunée G, Dellar M, Münger E, Krol L, Atama N, et al. Silent reservoirs are shaping disease emergence: the case of Usutu virus in the Netherlands [Internet]. 2024. Available from: http://biorxiv.org/lookup/doi/10.1101/2024.12.17.628855

12. de Wit MM, Martins AD, Delecroix C, Heesterbeek H, ten Bosch QA. Mechanistic models for West Nile virus transmission: a systematic review of features, aims and parametrization. Proceedings of the Royal Society B: Biological Sciences. 2024 Mar 13;291(2018).

13. Dakos V, Carpenter SR, Brock WA, Ellison AM, Guttal V, Ives AR, et al. Methods for detecting early warnings of critical transitions in time series illustrated using simulated ecological data. PLoS One. 2012;7(7).

14. Ebisuzaki W. A method to estimate the statistical significance of a correlation when the data are serially correlated. J Clim. 1997;10(9):2147–53.

15. Brett TS, O'Dea EB, Marty É, Miller PB, Park AW, Drake JM, et al. Anticipating epidemic transitions with imperfect data. PLoS Comput Biol. 2018;14(6):1–18.
