# Supplementary material for: Multivariate resilience indicators to anticipate vector-borne disease outbreaks: A West Nile virus case-study
Source: PLoS Comput Biol. 2025 Oct 13;21(10):e1012703. doi: 10.1371/journal.pcbi.1012703 (PMC12539738; doi:10.1371/journal.pcbi.1012703)
Supplement: Attachment — Submitted filename: Rebuttal letter2.pdf [file pcbi.1012703.s005.pdf]

Juliette Paireau
Academic Editor
PLOS Computational Biology

Roger Kouyos
Section Editor
PLOS Computational Biology

24-09-2025

Rebuttal letter of manuscript titled 'Multivariate resilience indicators to anticipate vector-borne disease outbreaks: a West Nile virus case-study'

Dear Editors,

We thank the reviewers for their generous comments on the previous version of the manuscript. We have now addressed their concerns in a revised version. In particular, we solved the unclarities pointed out by the reviewers and included more of our previous answers in the manuscript.

With kind regards,

Clara Delecroix,
On behalf of all authors.

Reviewers Comments:

Reviewer #1: Thank you for addressing all my previous comments and concerns

Thank you for revising this manuscript, we are happy to hear that you are satisfied with the changes made.

Reviewer #2: Thank you for your detailed, point by point response. All my previous concerns have been fully adressed.
I have only one minor comment: l364 the scenario with the 0.56 AUC seems to correspond to the Anthro-Equine scenario with explained variance. Please double check and correct if necessary.

Thank you for your comment, and for pointing out that mistake. We have updated the manuscript accordingly.

Reviewer #3: Review of PCOMBIOL-D-25-012124_R1

The rebuttal letter is adequate, and this version of the manuscript is improved. I have one major suggestion which is to incorporate more of the authors' responses to the reviewers into the manuscript. For instance, Reviewer #2 section 6 questions for authors. Readers may have these questions as well.

Thank you for reviewing our manuscript. We have included the following responses in the manuscript:

- *Previous comment from reviewer (C):* *Figure 1: Is it expected behavior for bird hosts to have higher dead incidence than seroprevalence at certain points in the simulation (e.g. panel C near the end)?*

Reply in the previous rebuttal letter (R): *For the visible bird species, 30% of infected birds die, leading to a low seroprevalence of this species. Additionally, in the compartment of recovered birds decreases because of natural death of the birds, whereas the compartment of dead birds only increases over time.*

Included lines 151-153: "*Bird species V is a visible, i.e. detectable, reservoir for the disease: 30% of the infected individuals die from infection, leading to high number of dead birds but low seroprevalence (as can be the case for corvids, for instance (30,34)).*"

And lines 168-173: "*The bird and horse populations are assumed to be at equilibrium in the absence of disease as the natural birth rate and the natural death rate (respectively b_B=m_B and b_E=m_E) are considered equal. The natural death rate affects all compartments of birds and horses equally. They are affected by an additional disease-induced death rate v_{VA}, $v_{HB,}$ and $v_{E,}$ respectively, and the count of dead birds V over time is reported in a compartment B_{VD}, which is the cumulative number of dead birds V and thus only increases over time.*"

- *<u>C:</u> Figure 3, 4, 5: Could you confirm whether each AUC points represent the classification performance across 200 simulations (100 with increasing R0 and 100 with stable R0) for each cut-off of Kendall tau?*

<u>R:</u> The AUC are indeed calculated across 200 simulations, split equally between data with upcoming epidemic and data without an upcoming epidemic.

Included lines 311-315: "*The ROC curve is calculated based on each value of Kendall tau from the 100 simulations with an upcoming epidemic and 100 simulations with no upcoming epidemic. Each value of Kendall tau is labeled with the outcome (upcoming epidemic or not), and the ROC curve is implemented using the perfcurve function of the Statistics and Machine Learning toolbox in Matlab.*"

- *<u>C:</u> Figure 5B: Could you explain what variable changes between points the same data type and species (for example the several blue triangles)?*

<u>R:</u> The change in the typical infection coefficient is simulated as a change of biting preference toward bird V. However, for horses that are already unpreferred species also not densely present, the change of biting preference towards the birds does not lead to a great change in their typical infection coefficient, hence the several almost overlapping blue triangles.

Included lines 256-258: "*The feeding preference coefficient affected the typical infection coefficient of all species differently, for instance it made little difference for horses as they are not highly abundant (Fig S12).*"

- *<u>C:</u> Given that you maintain the R0 within 0.7 – 1.0 by adjusting biting rates when varying mosquito feeding preference towards hidden birds, have you verified that bite rate remain biologically plausible under extreme preference scenarios?*

<u>R:</u> Thank you for pointing that out. The biting rate remains in a plausible interval (from 0.2 to 0.92), consistent with WNV parameter values retrieved in (12).

Included line 211 "*The biting rate varies over time (staying in the plausible interval 0.2-0.92 suggested in (35)), altering the R_0 and thus the resilience of the system.*"

- We treated 3 comments of reviewer 3 together, by including Figure I of the previous response to reviewers in the Supplementary Information (Figure S30 + text).

<u>C (1/3):</u> Line 416: The authors stated that « The prediction performance of the different multivariate monitoring scenarios, as well as univariate time series, remained stable regardless of how much a species is involved in the transmission cycle. Noticeably, increasing the mosquito feeding preference towards a given species or the relative abundances of the different species did not increase the potential for that species to signal upcoming outbreaks » which they say contradict earlier findings. However, the explanation provided -high spillover rates to dead-end species- appears inadequate. Looking at Figure 5A and S13, the performance of univariate variance-based RI is comparable across different species regardless of incidence (e.g. horse incidence). Moreover, this hypothesis can be tested with further analysis, such as sensitivity analysis of human-or-horses-based RIs performance with respect to these parameters. Therefore, further investigation is needed to fully understand this result.

<u>R (1/3):</u> Thank you for your comment. We agree that this result was surprising, and despite running some additional analyses (that we did not include in the manuscript) to investigate if some properties of the time series drive the prediction performance of resilience indicators, we could not find a satisfactory explanation (Figure I).

These analyses were run similarly to the analyses presented in the manuscript for Figure 5B: calculating the AUC of the univariate indicators for the different univariate time series generated

when varying the biting preference of the mosquitoes towards the hidden bird reservoir. However, here we investigate the effect of intrinsic properties of the time series: the number of zeros in the time series (Figure I A and B), the amplitude of the time series, calculated as maximum y – minimum y (Figure I C and D), the maximum value in the time series (Figure I E and F) and the sum of all the values in the time series (Figure I G and H).

For instance, a species with a relatively low involvement in the transmission cycle would exhibit a high number of zeros due to the rare observation of cases (Figure I A and B), whereas a species with a high involvement will exhibit a high maximum in the time series (Figure I E and F) and a larger amplitude (Figure I C and D). However, none of these processes seem to influence the prediction performance quantified using the AUC. Further analyses are required to understand what really drives how informative a data source is.

C (2/3): For variance as an indicator, how does the approach deal with mean-variance scaling? How does this approach perform at different population sizes? What happens to the differential performance of multivariate versus univariate resilience indicators as population sizes become very small or very large? (By manipulating population size, I mean manipulating the overall magnitude of the time series.)
R (2/3): Indeed, many things can have an effect on variance, for instance the amount of noise, whether it is autocorrelated and the mean value (if the noise is multiplicative). Therefore we only compare the indicators within the same time series, by calculating the correlation with time, where we assume that these effects stay constant. Moreover we conducted a sensitivity analysis varying the relative abundances of bird V and bird H, influencing the magnitude of the time series (Supplementary Material, Figures S21 to S25). In our case, the results were consistent across the tested population abundances.
Additionally, as specified in the response to the last minor comment of reviewer 2, exploratory analyses found no effect of the amplitude of the time series on the prediction performance of univariate indicators (Figure I).

C (3/3): Figures 4 and 5: I'm not sure how to interpret changes in AUC as a function of variables such as sampling resolution and feeding preference. Why do AUC values fluctuate—for example, increasing slightly rather than decreasing—when sampling resolution goes from weekly to biweekly? When should such fluctuations be interpreted as meaningful trends, and when are they considered negligible? This part of the analysis may require a clearer statistical framework and potentially more replicate simulations.
R (3/3): Thank you for your comment. Due to time constraints and limited computational power, we decided to stick with 200 replicates (100 emergence time series and 100 stable time series) throughout the analysis, as it yielded robust results across the different analyses. As stated in the text, further research is necessary to find what drives how informative a data source is, and what could explain the fluctuations observed in Figure 4 and 5. Additionally, as stated in a similar comment of reviewer #2, additional explorative analyses (not included in the manuscript) could not provide answers to that question. Although we think this is an important question, our analyses only provide initial answers (what does not drive how informative a source is). We think

however that this is an important result as it contradicts previous findings, and leaves room for further research dedicated to that question.

Lines 448-451: "Thus, the variability in the prediction performance of the different monitoring scenarios could not be explained by the typical infection coefficient, namely how much a species gets infected by a typical infectious individual (Fig 5B), nor by additional properties of the time series (Figure S30)."

Minor comments:

Line 99 does this phrase need a comma? "mean, autocorrelation and variance"

We mean the indicator "mean autocorrelation". We have rephrased the sentence to improve its readability "*for instance, mean autocorrelation and mean variance*"

Line 291 a window width of 50% the length of the time series seems rather large. Could the authors remark here on: 1) why they made it so wide and 2) how they handled missingness when the window extended beyond the beginning or end of the time series, which would occur often given the wide window?

Thank you for pointing out that unclarity. We do not handle missing data as we use only complete windows.

Lines 276-282: "*A rolling window of 50% of the size of the detrended time series is used to calculate the indicators and assess their trend over time, reflecting the most common choice for the size of the rolling window (2). All complete windows are used, with the first spanning from the start to the midpoint of the dataset, and the last spanning from the midpoint to the end. In each window, the value of each indicator is calculated (for univariate indicators, using the built-in functions to calculate variance and autocorrelation in Matlab) (7).*"

Line 314 This is not correct for PCA. Suggest: "The maximum eigenvalue of the covariance matrix used in PCA is equal to the amount of variance explained by the first principal component."

We have updated the manuscript accordingly.

Lines 299-301: "*The maximum eigenvalue of the covariance matrix used in the PCA is equal to the amount of variance explained by the first principal component (expl var).* "
