# Supplementary material for: Multivariate resilience indicators to anticipate vector-borne disease outbreaks: A West Nile virus case-study
Source: PLoS Comput Biol. 2025 Oct 13;21(10):e1012703. doi: 10.1371/journal.pcbi.1012703 (PMC12539738; doi:10.1371/journal.pcbi.1012703)
Supplement: Attachment — Submitted filename: review_report_PCOMPBIOL-D-24-02124.docx [file pcbi.1012703.s002.docx]

**Review report** – Multivariate resilience indicators to anticipate vector-borne disease outbreaks: a West Nile virus case-study.

1. **Article summary**

The article examines and evaluates various resilience indicators (RIs), which are statistical indicators used to detect early warning signs of critical transitions in complex systems, in the context of epidemiological surveillance. The study focuses on the application of RIs to vector-borne diseases, with the goal of improving early warning systems for disease outbreaks. To achieve this, the authors conduct a simulation study on West Nile Virus, a mosquito-borne virus that is expected to become more prevalent in Europe due to favorable climate change conditions. The authors aim to assess the performance of RIs, which are based on the idea that systems approaching a tipping point will exhibit slowing recovery times i.e. it will take longer to return to its normal state after a perturbation. In the context of disease epidemiology, this means that RIs can potentially detect early warning signs of an outbreak emergence, which occurs when the basic reproduction number (R0) approach 1.

The authors demonstrate that in this case-study variance-based RIs outperform autocorrelation-based ones, and that multivariate RIs are more effective, especially when dealing with low-resolution data. Contrary to previous findings, the authors show that in this case study, there is no evidence to suggest that one species is more informative than another for early warning purposes, despite their respective contributions to the transmission cycle.

2. **General assessment:**

As someone who was not familiar with RIs and critical slowing down theory prior to reading this article, I found the authors’ explanation and application of these concepts clear and well-motivated. After conducting some brief review of the literature and analyzing the provided information, in my opinion the article is a well-structured and well-written research paper that provides an original contribution to the field regarding the use and evaluation of resilience indicators as a model-free tool for early warning systems in the context of West Nile Virus and, more broadly, vector-borne diseases. Through their analysis, the authors effectively demonstrate novel results: that variance-based RIs outperform autocorrelation-based ones, multivariate RIs outperform univariate indicators and are more robust to low-resolution data. Moreover, the authors showed that no species in this system is more informative for early warnings whether it is more involved in the transmission cycle. These findings have significant implications for real-life applications, such as the choice of strategy for data collection in the setup of an early warning system for West Nile Virus outbreak surveillance. For instance, the use of multivariate RIs could enable more effective surveillance, ultimately leading to improved disease outbreak prevention and control.

However, some aspects of the article require clarification or improvement, particularly regarding the precise methodology used to evaluate the performance of the RIs i.e. the classification of positive outbreaks and negative outbreaks in the computation of the AUC and at which temporal horizon. Providing more detailed information on this aspect would strengthen the article and enhance its overall validity.

3. **Strengths:**

- The paper is clear and didactically written, facilitating understanding.
- Figures are neat, easy to interpret and visually appealing.
- The use of a simulation study is particularly relevant in this context, allowing precise control over the elements of the epidemiological system. This approach enables an insightful exploration on how the different RIs perform across the different scenarios and data resolutions.
- The methodology used, rooted in critical slowing down theory seems robust and have been already validated.
- Scenario simulations with varying data streams and resolutions effectively illustrate that multivariate indicators outperform univariate indicators, especially in conditions of low data resolution. This result has significant implications for practical surveillance and supports initiating discussions on real-world implementations of surveillance strategies for early warning system.

4. **Detailed comments:**

Major comments:

- It appears that a crucial methodological detail is missing regarding the evaluation of RIs’ performance. While the authors state they used the AUC of the ROC curve based on the classification of true positives and true negatives at different Kendall tau cut-off values derived from simulation of emergence time series (upcoming epidemic vs. no upcoming epidemic), the article lacks specifics on how this classification was precisely conducted. Clarification is needed on how the data was generated, what decision rules were applied, and what temporal horizon was used for outbreak predictions. Without this information, it is difficult to assess the validity and practical applicability of the RIs, particularly in the context of an early warning system.

Minor comments:

- The introduction could gain clarity from minor structuring. For example, the authors introduced West Nile Virus on Line 79, but reintroduce it Line 114. Additionally, on Line 98 the authors stated that multivariate indicators have not been compared to univariate indicators, and they restate the same claim Line 111.
- The « data poor » scenarios assume regular sampling intervals. It would be beneficial, at least briefly, to discuss or assess how irregular sampling (data points missing-at-random) or varying sampling frequencies across hosts (in multivariate indicators settings) might impact RIs performances.
- Line 337: the authors stated that autocorrelation based RIs perform worse than variance one due to the scarcity of cases resulting in highly variable autocorrelation due to long stretches of 0. Looking at figure 1, I see how it could be the case for horses for example but is this equally true for bird and mosquito incidence? This warrant clarification.
- Line 340: After the results of the performance of the RIs, authors choose to continue the analyses with the hidden bird reservoir incidence or seroprevalence and in the case of the multivariate RIs with the hidden reservoir scenario. Given that, by definition, this reservoir is hidden, evaluating observational probability or down sampling on this data source seems somewhat contradictory. Especially when you consider that for the three best performing multivariate RIs, the wildlife scenario is on par with the hidden reservoir scenario performance. Clarification of intent or reconsidering the data source maybe beneficial.
- Line 416: The authors stated that « The prediction performance of the different multivariate monitoring scenarios, as well as univariate time series, remained stable regardless of how much a species is involved in the transmission cycle. Noticeably, increasing the mosquito feeding preference towards a given species or the relative abundances of the different species did not increase the potential for that species to signal upcoming outbreaks » which they say contradict earlier findings. However, the explanation provided -high spillover rates to dead-end species- appears inadequate. Looking at Figure 5A and S13, the performance of univariate variance-based RI is comparable across different species regardless of incidence (e.g. horse incidence). Moreover, this hypothesis can be tested with further analysis, such as sensitivity analysis of human-or-horses-based RIs performance with respect to these parameters. Therefore, further investigation is needed to fully understand this result.

5. **Suggestions:**

- Figure 1: Panel readability could improve by decreasing panel A’s size and enlarging panel B and C because plots are difficult to read in these. Additionally, including plots of variance and autocorrelation of incidence or seroprevalence time series here might clarify RIs’ behaviors visually. Also, for the two bottom plots, the legends are not consistent between left and right plots.
- Figure 2: Clearly indicate the recovery time unit.
- Figure 3: Consider adding a y-axis for each panel or using a darker grid for improved readability.
- Line 343: Likely « human seroprevalence » rather than prevalence.
- Line 356 and 358: Panels should be ordered in the same sequence as they appear in the text i.e. 4A and 4B; 4C and 4D.
- Line 361: Rephrase the sentence to improve clarity, for example: « Multivariate indicators demonstrated greater robustness than univariate indicators, maintaining higher performance despite reductions in data resolution and observation probability »
- Line 442: Elaborate briefly on why system reactivity could influence variance performance.
- Line 444: This sentence in unclear especially considering the previous statement that several sources of data require increased collection efforts. It may need rephrasing to clearly communicate intended meaning.

6. **Questions for the authors:**

- Figure 1: Is it expected behavior for bird hosts to have higher dead incidence than seroprevalence at certain points in the simulation (e.g. panel C near the end)?
- Figure 3, 4, 5: Could you confirm whether each AUC points represent the classification performance across 200 simulations (100 with increasing R0 and 100 with stable R0) for each cut-off of Kendall tau?
- Figure 5B: Could you explain what variable changes between points the same data type and species (for example the several blue triangles)?
- Given that you maintain the R0 within 0.7 – 1.0 by adjusting biting rates when varying mosquito feeding preference towards hidden birds, have you verified that bite rate remain biologically plausible under extreme preference scenarios?

7. **Recommendations**:

Based on these comments, a minor revision is recommended to address methodological clarification (especially the RIs performance assessment methodology) and minor comments.
